# Videogame training increases clinical well-being, attention and hippocampal-prefrontal functional connectivity in patients with schizophrenia

**DOI:** 10.1038/s41398-024-02945-5

**Published:** 2024-05-28

**Authors:** Maxi Becker, Djo J. Fischer, Simone Kühn, Jürgen Gallinat

**Affiliations:** 1 University Medical Center Hamburg-Eppendorf, Clinic and Policlinic for Psychiatry and Psychotherapy, Martinistrasse 52, 20246 Hamburg, Germany; 2 Humboldt-University Berlin, Department of Psychology, Berlin, Germany; 3 Lise Meitner Group for Environmental Neuroscience, Max Planck Institute for Human Development, Berlin, Germany; 4 Max Planck-UCL Center for Computational Psychiatry and Ageing Research, Berlin, Germany

**Keywords:** Schizophrenia, Human behaviour, Predictive markers

## Abstract

Recent research shows that videogame training enhances neuronal plasticity and cognitive improvements in healthy individuals. As patients with schizophrenia exhibit reduced neuronal plasticity linked to cognitive deficits and symptoms, we investigated whether videogame-related cognitive improvements and plasticity changes extend to this population. In a training study, patients with schizophrenia and healthy controls were randomly assigned to 3D or 2D platformer videogame training or E-book reading (active control) for 8 weeks, 30 min daily. After training, both videogame conditions showed significant increases in sustained attention compared to the control condition, correlated with increased functional connectivity in a hippocampal-prefrontal network. Notably, patients trained with videogames mostly improved in negative symptoms, general psychopathology, and perceived mental health recovery. Videogames, incorporating initiative, goal setting and gratification, offer a training approach closer to real life than current psychiatric treatments. Our results provide initial evidence that they may represent a possible adjunct therapeutic intervention for complex mental disorders.

## Introduction

In the past years, scientific evidence has accumulated indicating that playing certain videogames has a positive impact on cognitive performance, as well as brain structure and functional connectivity (FC) in healthy participants [1, 2] (HP). Studies report performance improvements in different cognitive domains due to videogame interventions [3–6]. Above all, attention and visuospatial cognition were found to be most influenced by playing video games, particularly action videogames which is one type of game genre (see meta-analyses [7, 8]). In this context, it is of particular interest that videogame interventions have been shown to induce changes in plasticity in FC as well as in brain structure typically in the form of gray matter volume increases [1, 2, 9, 10]. Increases in FC have been observed after videogame interventions for task-related [11, 12] as well as resting-state connectivity measurements in HP [13]. For this reason, brain plasticity changes have been discussed as an important neurobiological mechanism for videogame-related improvement of cognitive performance [14].

The overall strong effects of videogames on brain plasticity and cognitive performance [15, 16] have been suggested to rely on general game demands. They refer to the continuous and goal-oriented interaction with the game including internal and external reinforcements (e.g. collecting credits, feeling of competence) accompanied by activating the dopaminergic reward system [3, 17, 18]. While general game demands require a broad mix of general cognitive functions [1] (attention, working memory, inhibition), the presence of spatial navigation in e.g. action-based videogames may constitute an additional important cognitive function with particular effects on hippocampal and entorhinal cortex plasticity [10, 14]. Engaging in action-oriented video games concurrently resulted in a growth of both hippocampal and prefrontal gray matter volume, alongside an enhancement in spatial orientation abilities (i.e., a shift in navigational strategy) [10]. Furthermore, theta range connectivity between both brain areas (PFC, HC) is highly relevant for spatial working memory [19] and attentional control, i.e. rapid detection of unexpected environmental events [20, 21]. In this context the question arises to what degree spatial navigation contributes to game-related brain plasticity and increased cognitive performance and whether this or rather general game demands could be of beneficial effect on cognitive performance and possibly also on psychopathology in psychiatric disorders [1].

One group of individuals possibly benefitting from the observed effects of action videogame interventions are patients with schizophrenia [22] due to prominent impairments in cognition [23–25] accompanied by positive (e.g. delusion and hallucinations) and negative symptoms (e.g. anhedonia and amotivation) [26]. Cognitive impairments in this disorder particularly comprise executive functions, attention and working memory [23–25, 27]. Of note, cognitive deficits and negative symptoms have the strongest impact on the patient’s functional outcome and are therefore also considered to be a core aspect of this disease [28].

The neural basis of these symptoms has been hypothesized to be the result of dysregulated neurotransmitters, such as glutamate and dopamine [29, 30], or altered structural brain plasticity, particularly in the Hippocampus (HC) and Prefrontal Cortex (PFC) [31–35]. One prominent hypothesis is, however, that disease related symptoms are best understood as plasticity aberrations of FC between different brain areas [36–40]. Evidence suggests that FC between HC and PFC in schizophrenic patients is reduced during resting-state [41, 42] and task-related fMRI measures [43]. This altered HC-PFC connectivity is associated with impairments in working memory performance [44, 45] and has therefore been discussed as a contributing factor for cognitive deficits but also negative symptoms in the disease [46–48]. Whereby pharmacological treatment options mostly target positive symptoms successfully, for negative symptoms and especially cognitive deficits less treatment options exist [49, 50]. Behavioral interventions, which have the potential to mitigate cognitive impairments and negative symptoms, could be a valuable supplementary therapeutic approach for individuals with schizophrenia.

In this study, we explore the possibility of transferring the positive training effects observed in HP to patients with schizophrenia through an eight-week video game training program specifically targeting the HC-PFC network. Due to well-established cognitive impairments and reduced neural plasticity in this patient group [51, 52], we anticipate reduced intervention effects compared to HP. For this purpose, we trained schizophrenia patients as well as HP with an action/platform videogame (APVG) with two different game mechanics: one APVG had a 2D game design (character moves only from left to right to achieve goals) and a highly similar APVG but with a 3D game design (character moves in 3-dimensions to achieve goals). Only the 3D game version includes the need to train spatial navigation abilities [10, 53]. Both videogame conditions (3D and 2D condition) were compared to an active control condition reading E-books (E-book condition, Amazon Kindle, 2014) over a period of eight weeks.

Regarding the composition of cognitive demands (with or without spatial navigation), we expect two possible outcomes. If increased spatial navigation is the primary training mechanism underlying the abovementioned effects, then we expect a training-related decrease in negative symptoms and an increase in cognition (specifically spatial orientation) together with associated HC-PFC FC for the 3D condition (3D > 2D > E-book). Should the more general game demands have the strongest training effect being approximately equally expressed in both videogames, we would expect a significant effect of both videogames compared to the E-book condition (3D/2D > E-book).

## Material and methods

The study was preregistered (https://clinicaltrials.gov/ct2/show/NCT03522220) and all further outcome measures not directly related to the present paper are reported in the Supplements.

### Participants

All participants were pre-screened to ensure exclusion of individuals reporting more than one-hour videogame usage per day in the past six months or prior exposure to *Super Mario 64* or *New Super Mario Bros*. In addition, the exclusion criteria for magnetic resonance imaging (MRI) measures were applied (see preregistration). The local ethics committee of the General Medical Council Hamburg, Germany approved the study (PV4974). Written informed consent of the participant or legal guardian was obtained after a detailed explanation of the study procedure and they received financial compensation after the study participation.

#### Patients

**Fig. 1:**
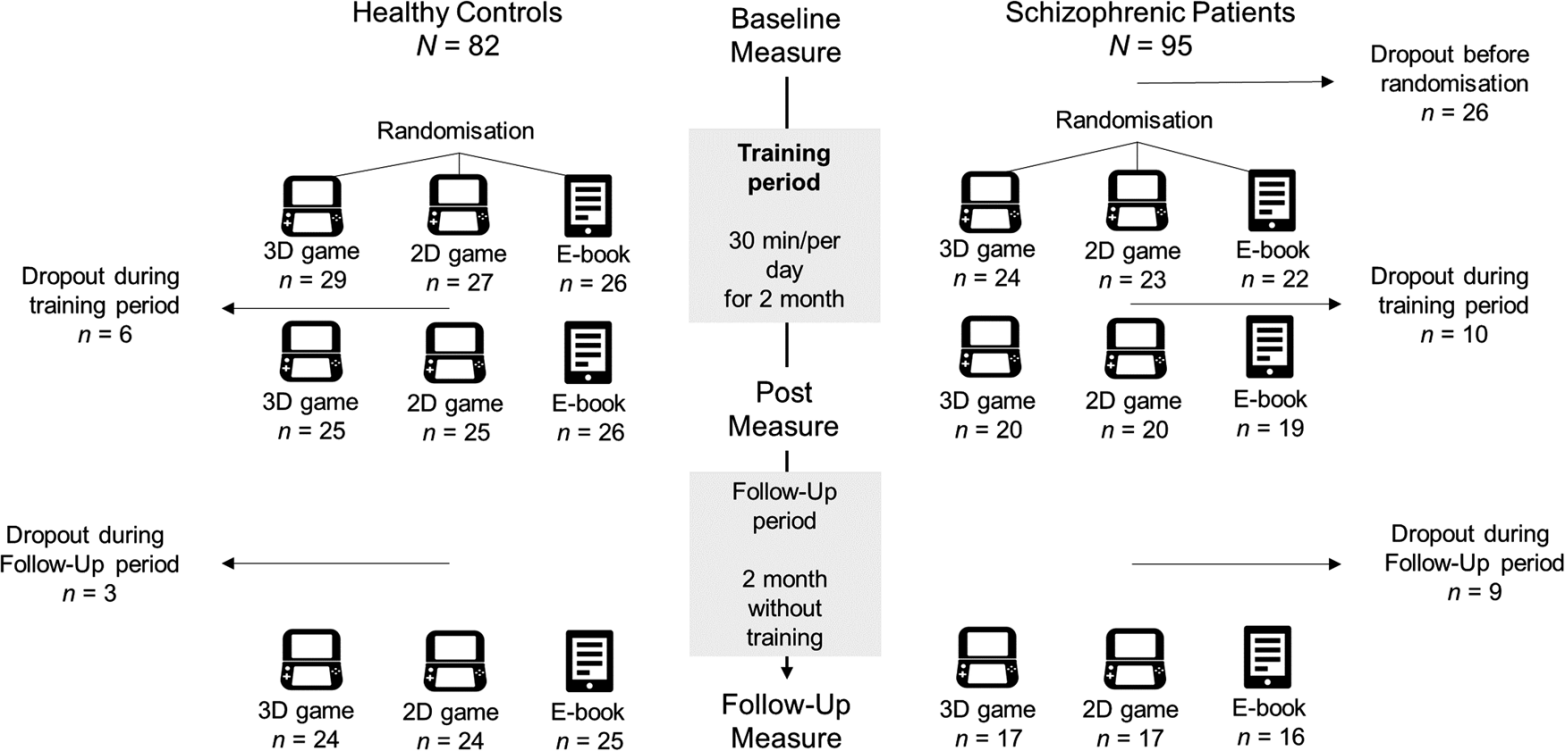
Study procedure and dropout. *Note*. 3D game = Super Mario 64; 2D game = Super Mario Bros.

Ninety-five patients with schizoaffective disorder or schizophrenia (henceforth patients) were recruited via the psychosis ward of the Psychiatry Department of the University Medical Center Hamburg-Eppendorf (Germany). Four trained interviewers rated a modified interview adapted from the German version of the Mini International Neuropsychiatric Interview (MINI [54]). Individuals not fulfilling the criteria for schizoaffective disorder or schizophrenia based on the International Statistical Classification of Diseases and Related Health Problems (ICD-10) were excluded, as well as individuals with other major psychiatric or neurological illnesses or significant alcohol or substance abuse in the past six month. The patients were clinically stabilised, but still showed residual symptoms, scoring at least 3 points or above on one item of the Positive and Negative Syndrome Scale (PANSS [55]). From 69 eligible patients, ten individuals dropped out during the training period (see Fig. 1), resulting in a final sample of *N* = 59 patients (*n* = 52 schizophrenia, *n* = 7 schizoaffective disorder, see Table 1).

**Table 1:** Demographic and clinical characteristics for each experimental group at baseline.

Baseline characteristic	Healthy controls			
	EB (*n* = 26)	2D (*n* = 25)	3D (*n* = 25)	Full sample (*N* = 76)
Age in years	29.92 (8.25)	29.56 (9.11)	30.24 (8.05)	29.91 (8.37)
Sex^1^	42%	44%	44%	43%
Educational years	16.80 (3.01)	16.06 (3.86)	16.41 (3.53)	16.43 (3.49)
MCCB	51.43 (9.78)	53.52 (7.29)	52.08 (8.95)	52.28 (8.81)
MCCB _Attention_	49.75 (10.58)	51.44 (8.70)	52.73 (11.45)	51.26 (10.40)

	Patients with schizophrenia			
	EB (*n* = 19)	2D (*n* = 20)	3D (*n* = 20)	Full sample (*N* = 59)
Age in years	31.84 (10.23)	34.00 (11.21)	32.40 (8.15)	32.76 (9.81)
Sex^1^	47%	45%	50%	47%
Educational years	16.08 (4.28)	16.40 (3.86)	15.78 (4.14)	16.08 (4.03)
MCCB	41.15 (9.43)	42.75 (12.08)	43.89 (9.99)	42.02 (10.71)
MCCB _Attention_	39.41 (10.91)	42.86 (11.94)	43.32 (11.36)	41.49 (11.30)
PANSS	58.64 (12.29)	53.34 (10.64)	52.77 (11.24)	54.86 (11.50)
PANSS_pos_	13.39 (4.40)	12.82 (3.42)	12.47 (3.79)	12.89 (3.83)
PANSS_neg_	14.49 (3.56)	13.40 (4.60)	13.12 (4.52)	13.66 (4.23)
PANSS_gen_	30.76 (6.72)	27.11 (5.60)	27.18 (5.94)	28.31 (6.22)
Age of illness onset	22.74 (8.53)	26.62 (8.52)	21.50 (5.69)	23.71 (7.92)
Medication dose^2^	13.13 (14.97)	12.97 (16.85)	12.49 (11.82)	12.86 (14.44)
Medication years	7.21 (8.09)	6.08 (5.73)	8.77 (7.46)	7.33 (7.10)
Number of episodes	8.92 (16.82)	3.97 (4.37)	10.92 (19.23)	7.94 (14.99)

Values represent means (standard deviations). ^1^per cent of female participants. ^2^Antipsychotic medication dose at baseline presented in Olanzapine equivalent dose based on defined daily doses [60]. *EB* E-book, *MCCB* total score (averaged for pre/post time points), *MCCB*_*Attention*_ Continuous Performance Task representing sustained attention (averaged for pre/post time points), *PANSS*_*pos*_ Positive Subscale, *PANSS*_*neg*_ Negative Subscale, *PANSS*_*gen*_ General Psychopathology Subscale; All presented *PANSS* scores include only the pre time point values, low values indicate reduced symptom severity.

#### Healthy participants

Through announcements and internet advertisements, eighty-two HP were recruited and screened using the modified MINI to exclude a psychiatric or neurological illness and matched with the patients concerning age, sex and education years. Removing the dropout of six HP during the training period (see Fig. 1), the final sample comprises *N* = 76 HP (see Table 1).

### Training procedure

**Fig. 2:**
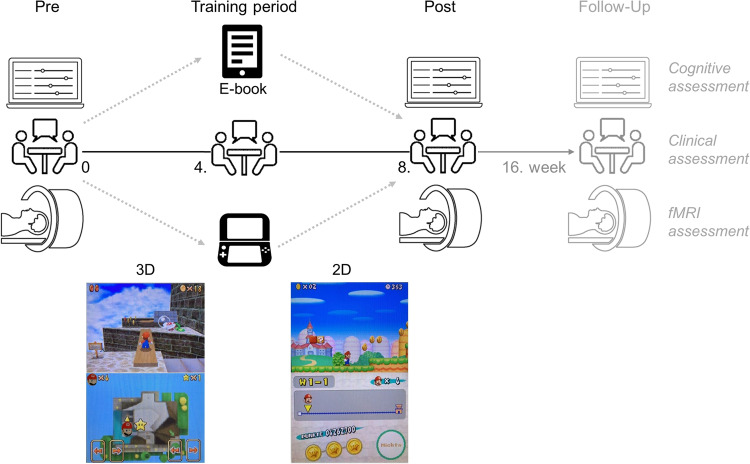
Videogame training procedure. 3D = Super Mario 64; 2D = New Super Mario Bros. Patients with schizophrenia *and* healthy controls were trained with either a videogame (3D or 2D condition, see screenshot) or an E-book (active control condition) for eight weeks. Before (Pre) and after (Post) the intervention all subjects underwent cognitive and fMRI assessments. The patients additionally underwent clinical assessments at each time point including during the 4. week of the intervention. We additionally assessed all patients eight weeks after the end of the intervention (Follow-Up) but were mostly interested in the immediate trainings effects (Post). Results for the Follow-Up time point are reported in the Supplements (see Fig. S2).

All patients and HP were randomly assigned to one training condition by a person who was not involved in the clinical interview in a between-subject design and were instructed to play or read for at least an average of 30 minutes per day over the 2-month training period (consistent with previous videogame interventions [10, 11]). The experimental groups played either the videogame Super Mario 64 (3D) or New Super Mario Bros. (2D) on a portable Nintendo Dual Screen XXL console (Nintendo DS, 2008, Kyoto, Japan) (see Fig. 2 and Supplements). The active control condition (E-book) received an Amazon Kindle with thirteen pre-installed books in German language to choose from (see Fig. 2). Quality control included a weekly online questionnaire assessing the daily amount of playing/reading time (on average: E-book=30.73 min, 2D = 32.25 min, 3D = 31.30 min) and the amount of acquired stars [11] reflecting the achieved game level for both videogame conditions (see section data exclusion). Note, the different game structure resulted in a different average of collected stars in both conditions: 3D: 28 stars (*SD* = 30, range=0–126); 2D: 5 stars (*SD* = 3, range=0–8).

### Behavioural assessments

#### Cognitive assessments

The *MATRICS Consensus Cognitive Battery* (MCCB) was used to assess cognitive performance especially in schizophrenic patients [56] including seven cognitive domains: sustained attention, speed of processing, working memory, verbal learning, visual learning, reasoning and problem solving, and social cognition (see Supplements).

To assess participants’ spatial orientation, we used the *Tunnel task* [57] where participants follow a simulated ride through a tunnel on a screen in first-person perspective with 1–3 turns (3 difficulty levels) and indicate the direction of the start position at the end of the ride. The spatial orientation measure is the angle deviance between the estimated and correct direction (see Supplements).

#### Clinical assessments

We used the PANSS including its three subscales Positive, Negative and General Psychopathology (peer-assessment [55]) and the Recovery Assessment Scale (RAS [58], German version, self-assessment) including its five subscales *Personal confidence and hope, Willingness to ask for help, Goal and success orientation, Reliance on others, Not dominated by symptoms* (see Supplements).

### Behavioural data analysis

Data was modelled using Linear Mixed Models [59] including subject as random intercept (see Table 2). Dependent variables included: MCCB total score, Tunnel task, PANSS total score including its three subscales and the RAS total score. For exploratory purposes, we additionally examined the five RAS subscores.

**Table 2:** Formula for Linear Mixed Models to predict behavioural outcome measures.

	Dependent variable	Formula for Linear Mixed Models
a.	PANSS/RAS	~ condition * time + medication + fun + (1|subjects) + ε
b.	MCCB	~ condition * time * group + fun + (1|subjects) + ε
c.	Tunnel task	~ condition * time * group * difficulty + fun + (1|subjects) + ε

condition = Three ordered intervention conditions (3D > 2D > E-book); time refers to pre vs. post intervention; fun = weekly questionnaire indicating pleasure while dealing with respective media device; group = patients or healthy controls; medication = Olanzapine equivalence doses of antipsychotic medication [60].

The respective condition, time (1st week=pre and 8th week=post intervention) as well their interaction (condition*time) and group (patients vs. HP), served as independent variables. Regarding the condition, we hypothesised a gradient in the strength of the effects over time from the 3D to 2D to the Kindle condition, especially for the Tunnel task measuring spatial orientation. Therefore, the condition variable was modelled as ordered factor (3D > 2D > Kindle). Note, the time point pre and post intervention was primarily analysed due to our interest in the immediate intervention effect (see Fig. S2 for a visual depiction of the condition*time effect until the 16^th^ week =follow-up). These variables served as covariates of no interest (for more information see Supplements): Olanzapine equivalence doses (henceforth *medication* variable [60]), the perceived fun using the respective media device (henceforth *fun* variable [10]) and the amount of turns reflecting the difficulty level in the Tunnel task (henceforth *difficulty)*. P-values for the hypothesised interaction terms (condition*time[*group*difficulty]) were computed using likelihood ratio tests. Effect sizes are reported via standardized beta coefficients including their 95% confidence intervals. The response rates were calculated by analysing the Percentage Change of all significant outcome variables, which can be found in the supplementary material (Table S4). To provide insight into the clinical relevance, we present response rates indicating a decrease of at least 50% in the PANSS total score. When modelling the subscores for the MCCB and RAS, p-values from the likelihood ratio tests were corrected for multiple comparison using Bonferroni correction [61] for those subscores where we did not have an a priori hypothesis.

#### Data exclusion

To ensure that only participants who engaged with the corresponding video game were included in the experimental conditions (3D, 2D), we excluded individuals from further analyses if they did not complete the first world of the respective game (2D: at least one star; 3D: at least two stars) throughout the entire training period. This led to the exclusion of six subjects in total (2D: *n* = 4; 3D: *n* = 2). The number of achieved stars in those APVG has been used as measure for game progress before [11]. Values of all dependent variables (except PANSS) deviating >3 SD from the mean were excluded. Note, PANSS scores were obtained via two raters and thus extreme values were not assumed to be measurement errors (see Supplements, Tables S1−S3).

### fMRI data assessment and analysis

#### fMRI data acquisition

Resting state functional (henceforth rs-fMRI) and structural images were collected on a Siemens Skyra 3 T scanner (Erlangen, Germany) using a standard 32-channel head coil. Functional images were collected during rest while participants were asked to fixate a cross on the center of the screen with their eyes, using a T2*-weighted echo planar imaging (EPI) sequence sensitive to blood oxygen level dependent (BOLD) contrast (TR = 2000ms; TE = 30 ms, image matrix = 72 × 72, voxel size = 3.0 mm × 3.0 mm × 3.0 mm, flip angle = 80°, FOV = 216 mm, 36 axial slices, 210 volumes). The structural images were obtained using a three-dimensional T1-weighted magnetization prepared gradient-echo sequence (MPRAGE) (repetition time = 2500 ms; echo time = 2.12 ms; TI = 1100 ms, acquisition matrix = 240 × 41 × 194, flip angle = 9˚; 0.8 × 0.8 × 0.94 mm voxel size).

#### Rs-fMRI preprocessing and denoising

Rs-fMRI data were preprocessed via CONN’s default preprocessing pipeline using the default settings. This includes functional realignment, slice time correction, as well as outlier identification of functional scans, normalisation of functional and structural scans into MNI space, segmentation and 8 mm smoothing of functional data (version 21.a [62]). Functional data were further denoised using CONN’s default denoising pipeline [62] and band-pass filtered to 0.008 - 0.09 Hz.

#### Rs-fMRI data analysis

##### First Level Analysis

Due to our specific hypothesis, we adopted a region-of-interest (ROI)-based approach creating a HC-PFC specific network. This network included PFC ROIs taken from CONN’s default network atlas [62] and bilateral HC ROIs taken from CONN’s default *FSL Harvard-Oxford atlas* (note, CONN’s network atlas does not include HC ROIs) [63]. Because we did not have a specific hypothesis which prefrontal ROIs may increase in FC with the HC, we used all 12 PFC ROIs available in this atlas. Each ROI’s time series was computed by (mean) averaging the BOLD time series of each voxel belonging to the respective ROI. ROI-to-ROI connectivity matrices (14 × 14) were computed separately for each participant and time point (pre, post) (*N* = 132).

##### Second Level Analysis

To analyse condition-induced changes over time in this HC-PFC network, a multivariate parametric General Linear Model (GLM) was performed for all 14 × 14 HC-PFC connections as implemented in CONN. First, we analysed all subjects together to investigate an overall condition*time effect on FC controlling for *group* (patients or HP) and experienced *fun* using a media device (see behavioural data analysis). The between-subject contrast was set to -1.5*Kindle + 0.5*2D + 1*3D + 0*group + 0*fun and the within-subject contrast was set to −1*pre + 1*post intervention. Note, we weighted the 2D condition with 0.5 instead of 0 to account for the finding from the behavioural data analysis that both gaming conditions performed similarly but differed from the active control condition (Kindle) while still keeping the order according to our previous hypothesis 3D > 2D > Kindle. For statistical inference, we adopted the Network-based Statistics (NBS) approach only including thresholded positive connections at *p* < 0.05 (uncorrected) because we had a directed hypothesis [64] and thresholded cluster (*p* < .05 FDR-corrected) at the network level. Finally, difference FC values between the pre and post intervention time point per HC-PFC connection were correlated with cognition to investigate possible APVG-related changes (for more detailed information see Supplements).

## Results

Standardized parameter estimates for all individual predictors are listed in Tables S1–S3. Here we concentrate on reporting the results of the assumed interaction effects and respective Posthoc tests.

### Effects of training intervention on spatial orientation and cognition

Based on prior meta-analyses [7, 8], we assumed training-induced changes in spatial cognition and sustained attention.

#### Tunnel task

**Fig. 3:**
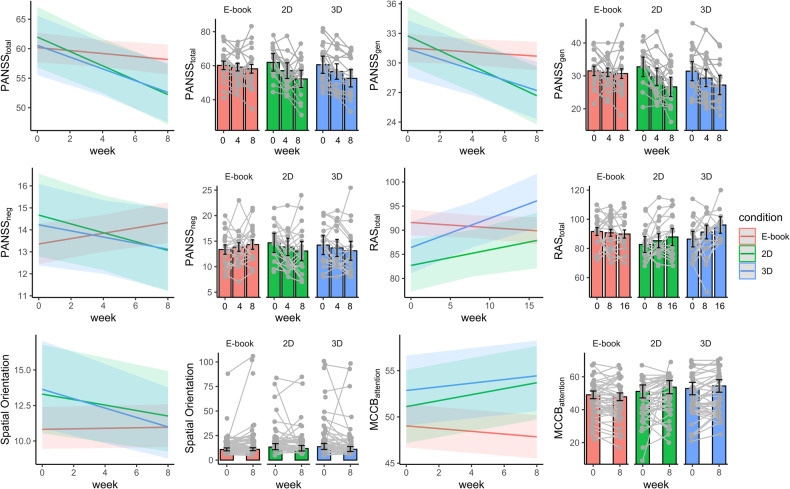
Intervention-induced increase in cognition and clinical well-being. PANSS_gen_ = General Psychopathology Subscale, PANSS_neg_= Negative Subscale, values represent estimated marginal means. For all presented *PANSS* scores, low values indicate reduced symptom severity. Higher scores in the *RAS*_*total*_ indicate higher perceived mental health recovery. *Spatial Orientation* shows the angle deviance between the estimated and correct direction, with lower values indicating better performance. For *MCCB*_*Attention*_ higher scores indicate better performance in the cognitive domain of attention. Upper panel: Coloured shades around regression lines represent 95% between-subject confidence intervals. Lower panel: Bar graphs illustrate estimated marginal means for each outcome variable, split by time and condition. These graphs feature overlaid individual (raw) data points and their changes over time. Error bars denote 95% between-subject confidence intervals.

The condition*time interaction in the likelihood ratio test was not significant (*χ²*(2) = 5.35, *p* = 0.068) but showed a significant linear trend in the hypothesized direction *t(*628.93) = −2.30, *p* = 0.021; ß = −0.05, *CI*[−0.02, −0.08]). The group in the 3D condition performed better (i.e., less angle deviance) than the E-book condition (*t*(628.8) = −2.30, *p* = 0.021; ß = −0.07, *CI*[−0.02, −0.12]) but not better than the 2D (*p* > 0.35) condition while the 2D and the E-book condition also did not differ in their performance (*p* > 0.16) (see Fig. 3). No main effect for group (*p* > 0.24) and no three-way (*p* > 0.35) nor four-way interaction (*p* > 0.18) was observed.

#### MCCB

No condition*time interaction (*p* > 0.60) nor condition*time*group interaction (*p* > 0.20) was observed for the overall MCCB score but HP performed significantly better than patients (*t*(134.39) = 6.43, *p* < 0.001; ß=0.94, *CI*[0.65, 1.22]). The *Continuous Performance Test* (CPT) measuring sustained attention showed a condition*time interaction (*χ²*(2) = 7.58, *p* = 0.022) indicating a linear trend for condition over time (*t*(134.39) = 2.01, *p* = 0.046; ß = 0.08, *CI*[0.00, 0.17]). Posthoc analyses revealed that the 3D condition (*t*(128.1) = 2.01, *p* = 0.047; ß = 0.12, *CI*[0.00, 0.24]) as well as the 2D condition (*t*(127.9) = 2.68, *p* = 0.008; ß = 0.16, *CI*[0.04, 0.28]) performed significantly better in the CPT over time compared to the E-book while both videogame conditions did not differ significantly from each other (*p* > 0.48). Furthermore, HP performed significantly better than patients (*t*(133.7) = 5.89, *p* < 0.001; ß = 0.87, *CI*[0.58, 1.16]). The non-significant three-way interaction (*p* > 0.13) indicated that the condition*time effect in this domain did not differ between patients and HP. To rule out the possibility that merely enhanced proficiency with digital devices, hence improved reaction times, accounted for the observed improvements in sustained attention (notably, the CPT was the sole computer-based test in the MCCB), we examined whether both video game conditions compared to the control condition resulted in shorter reaction times for hits in this task. No evidence was found for a condition*time (Chi²(2) = 1.013, *p* = 0.602) nor condition*time*group (Chi²(5) = 2.62, *p* = 0.758) interaction for reaction time in this task, suggesting no evidence for improved reaction time in both videogame groups compared to the Kindle group in the CPT.

### Effects of training intervention on clinical symptoms

#### PANSS

The condition*time interaction for the PANSS_total_ score (sum score of all 30 items) was significant (*χ²*(2) = 7.77, *p* = 0.021) indicating a linear trend for condition over the time (*t*(121.9) = −2.11, *p* = 0.037; ß = −0.16, *CI*[−0.01, −0.31]). Post hoc analyses revealed a significant reduction of the PANSS_total_ score in the 3D compared to the E-book condition (*t*(121.9) = −2.11, *p* = 0.037; ß = −0.23, *CI*[−0.01, −0.44]) but not compared to the 2D condition (*p* > 0.52). Symptomatology was also more strongly reduced over time in the 2D compared to the E-book condition (*t*(123.6) = −2.73, *p* = 0.007; ß = −0.30, *CI*[−0.08, −0.51]) (see Fig. 3). Response rates of minimum 50% PANSS reduction were observed in 75% (*n* = 15) of the patients training in the 3D condition and 70% (*n* = 14) in the 2D condition, compared with 55% (*n* = 11) reading E-book (see Table S4).

Further investigation revealed that the effect in the PANSS_total_ score was driven by PANSS_gen_ and PANSS_neg_ scores: For the PANSS_gen_, we found a significant condition*time interaction (*χ²*(2) = 10.01, *p* = 0.006) indicating a linear trend for condition over time (*t*(121.4) = −2.09, *p* = 0.038; ß = −0.16, *CI*[−0.01, −0.31]). Posthoc analyses revealed that the 3D condition (*t*(121.4) = −2.09, *p* = 0.038; ß = −0.23, *CI*[−0.01, −0.44]) and the 2D condition (*t*(123.1) = −3.21, *p* = 0.002; ß = −0.35, *CI*[−0.13,−0.57]) exhibited less symptoms on this scale over time than the E-book condition but both videogame conditions did not significantly differ from each other (*p* > 0.24) (see Fig. 3).

A significant condition*time interaction (*χ*²(2) = 7.04, *p* = 0.029) was also found for the PANSS_neg_ score indicating a linear trend for condition over time (*t*(122.2) = −2.07, *p* = 0.040; ß = −0.16, *CI*[−0.01, −0.31]). Posthoc analyses revealed that the 3D (*t*(122.2) = −2.07, *p* = 0.040; ß = −0.23, *CI*[−0.01, −0.44]) and 2D (*t*(123.9) = −2.55, *p* = 0.012; ß = −0.28, *CI*[−0.06, −0.50]) condition exhibited a significant reduction of negative symptoms over time compared to the E-book condition but both video gaming groups did not significantly differ from each other (*p* > 0.61).

For the PANSS_pos_ score there was no evidence for a condition*time interaction (*p* > 0.77).

#### RAS

No condition*time interaction was observed (*p* > 0.58) for the RAS_total_ score. However, the fun variable, i.e. the extent of experienced pleasure while spending time with the respective media device, significantly predicted the amount of averaged self-assessed general recovery over both time points (*t*(60.78) = 4.11, *p* < 0.001; ß = 0.41, *CI*[0.21, 0.60]).

As mental health recovery might not be immediately apparent to patients after the training period, we explored delayed effects on mental health recovery by including a follow-up time point eight weeks after the intervention, allowing for modeling of pre, post, and follow-up periods to assess the hypothesized condition*time interaction. There was a significant condition*time interaction (*χ*²(2) = 10.29, *p* = .006) indicating a linear trend for the condition over the time course of sixteen weeks for the total RAS_total_ score (*t*(111.4) = 3.24, *p* = 0.001; ß = 0.26, *CI*[0.10, 0.42]). The subscores *Reliance on others (χ*²(2) = 6.10, *p* = 0.047; ß = 0.22, *CI*[0.04, 0.39]*), Personal Confidence and hope* (*χ*²(2) = 8.59, *p* = 0.013; ß=0.18, *CI*[0.03, 0.32]) and *Goal and Success Orientation* (*χ*²(2) = 10.97, *p*-Bonferroni = 0.021; ß = 0.29, *CI*[0.12, 0.46]) drove those linear condition*time effects of the RAS at follow-up. However, only *Goal and Success Orientation* survived correction for multiple comparison (see Fig. 3).

### Effects of training intervention on HC-PFC resting state FC

**Fig. 4:**
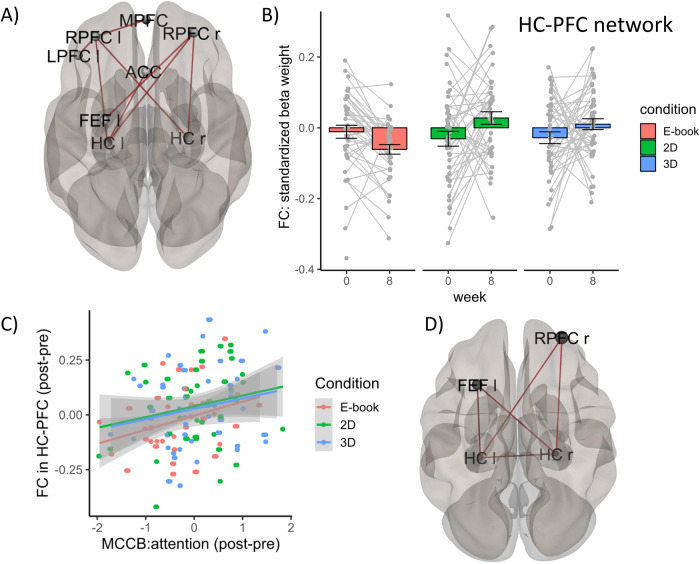
Intervention-induced increase in functional connectivity (FC) in a hippocampal-prefrontal (HC-PFC) network over time. **A** Significant nodes in HC-PFC network that increased in functional connectivity (FC) as a function of intervention over time (3D > 2D > E-book). FEF = Frontal Eye Field, RPFC = Rostral Prefrontal Cortex, MPFC = Medial Prefrontal Cortex, HC = Hippocampus, ACC = Anterior Cingulate Cortex, LPFC = Lateral Prefrontal Cortex. **B** Effect size for average FC values in HC-PFC network as demonstrated in (**A**) split for condition and time (in weeks). Barplots represent means ± SEM. **C**, **D** Significant positive correlation between intervention-induced FC changes in bilateral HC and right RPFC & left FEF in sustained attention (MCCB test battery) over time. Note, values = difference values (post - pre intervention). Grey shaded areas = standard error.

As predicted, we found evidence for an intervention-based increase (i.e. a significant condition [3D > 2D > Kindle] * time [Post>Pre] interaction) in connectivity between the Hippocampus (HC) and Prefrontal Cortex (PFC) (see Fig. 4). For this interaction, the ROI-to-ROI connectivity analysis revealed a significant PFC-HC network (Mass=94.47, *p*-FDR = .033) comprising bilateral HC, bilateral Rostral Prefrontal Cortex (RPFC), left Lateral Prefrontal Cortex (LPFC), Medial Prefrontal Cortex (MPFC), Anterior Cingulate Cortex (ACC) and left Frontal Eye Field (FEF). Within this network, the left HC exhibited increased FC with the left (*t*(127) = 2.65, *p* = 0.004) and right RPFC (*t*(127) = 2.44, *p* = 0.008), ACC (*t*(127) = 2.45, *p* = 0.008). Connectivity was also increased between the right HC and left RPFC (*t*(127) = 3.10, *p* = 0.001), and right RPFC (*t*(127) = 1.92, *p* = 0.029). The statistics for all connections can be found in Table S5.

However, no evidence for significant differences (*t*(130) = 1.84, *p* = 0.068; ß=0.32, *CI*[−0.02, 0.67]) in FC for this HC-PFC network between patients (*M* = −0.002, *SD* = 0.13) and HP (*M* = −0.04, *SD* = 0.12) was found during baseline (before the training intervention), suggesting no particular dysconnectivity for this particular network in this patient sample. For exploratory purposes, we investigated whether patients and HP contributed differently to those HC-PFC connections. When investigating the individual links between bilateral HC and PFC-ROIs, we found that HP (*N* = 76) showed most condition-induced (3D > 2D > Kindle) increase in FC between bilateral HC and left RPFC (*t*(73) = 2.58, *p*-uncor = 0.011, ß=0.19) and right RPFC (*t*(73) = 2.39, *p*-uncor = 0.019, ß=0.19) over time while patients (*N* = 54) showed most condition-induced (3D > 2D>Kindle) increase over time between bilateral HC and left RPFC (*t*(51) = 2.10, *p*-uncor = 0.040, ß=0.27) as well as ACC (*t*(51) = 2.34, *p*-uncor = 0.023, ß = 0.30) in addition to left IFG (*t*(51) = 2.42, *p*-uncor = 0.019, ß=0.22) and Anterior Insula (*t*(51) = 2.20, *p*-uncor = 0.033, ß=0.21) that were not part of the general HC-PFC network over both groups.

### Link between condition-induced changes in HC-PFC resting state FC and cognition

Ultimately, we investigated whether the changes in HC-PFC FC induced by the videogame condition are associated with improvement in cognitive performance (sustained attention and [to a lesser degree] spatial orientation) observed as a result of the condition. The cognitive domain sustained attention of the MCCB (CPT) correlated with increased FC in bilateral HC and right RPFC (*F*(2,127) = 7.37, *p*-FDR = 0.011, ß = 0.08) as well as left FEF (*F*(2,127) = 5.06, *p*-FDR = 0.046, ß=0.05) (see Fig. 4). All other variables did not survive multiple comparison (*p* > 0.36).

## Discussion

In this study, we provide evidence that training with an APVG over eight weeks compared to an active control increased performance in sustained attention which was additionally correlated with an increase of FC in the HC-PFC-network. Importantly, the present study used an active control condition (kindle reading) ruling out placebo effects in the intervention group to be responsible for the observed improvement. Moreover, spatial orientation showed no significant training effect but a descriptive increase in the hypothesised direction (*p* = 0.056). We found no evidence for intervention-related improvements in other cognitive subdomains. Importantly, patients showed a significant reduction in the PANSS total score driven by less negative symptoms and general psychopathology due to the APVG intervention including improvements of self-assessed mental health recovery (RAS) during follow up. Across all outcomes, no differences for the 2D versus 3D condition were observed, further suggesting a subordinate role of spatial navigation as a training mechanism.

### Effects of video game training on cognition and HC-PFC networks

The specific training-related effects on sustained attention and the descriptive trend for spatial cognition observed in the present study are in line with results from meta-analyses reporting those two cognitive domains to be most influenced by action videogaming in HP [7, 8]. Importantly, the training effect of the two APVGs did not significantly differ between patients and HP. However, the lack of evidence for a training mechanism relying on spatial navigation in the game as previously assumed [10, 65] suggests that the observed beneficial effects are primarily due to general game demands involving a continuous and goal-oriented interaction with the game including incentives and gamification elements. Similarly, other videogame studies in HP (e.g., Rise of Nation or Call of Duty) also found cognitive improvements despite varying demands on spatial navigation [4, 66–68].

Our findings provide first evidence that FC in a specific HC-PFC network increases due to the videogame intervention (note, we did not replicate earlier findings of training-induced structural plasticity [10, 53], for a discussion see Supplements). Certain HC-PFC connections (i.e. nodes between HC and the Salience [RPFC] and Dorsal Attention [FEF] network) were correlated to sustained attention performance confirming previous findings that the interaction between HC-PFC areas is involved in attentional control [20, 21] and can be targeted with an APVG intervention [12, 13]. This is of particular significance since disrupted HC-PFC connectivity has been consistently linked to cognitive impairment in schizophrenia [44, 69–72]. Additionally, addressing cognitive deficits [73], which are a core symptom of schizophrenia, proves challenging with antipsychotic treatments [50, 74]. However, it is important to note that no specific dysconnectivity pattern was detected in our patient group during baseline, indicating that the APVG intervention typically enhances HC-PFC FC rather than counteracting a patient specific deficit. This aligns with our observation that there were generally no group differences in cognitive improvements related to the intervention. Nevertheless, considering the consistent intervention-driven increase in HC-PFC FC seen in both schizophrenia patients and HP, future research may investigate the extent to which this training could enhance FC in individuals with pre-existing hypoconnectivity within this network, potentially alleviating specific deficits in FC.

### Effects of APVG intervention on psychopathology and recovery

Besides cognitive and brain effects, the intervention with APVG was associated with a severity reduction of psychopathology, namely the PANSS general score comprising depression, anxiety, and disturbance of volition among others. So what causes symptom reductions observed in the present study? Although speculative, the goal-driven and continuous interaction with the game may impact the patients’ experienced self-efficacy (defined as beliefs of how well one masters tasks and deals with prospective situations [75]) which has been linked to mental health and psychopathology [76–79]. The findings from the RAS are in line with this idea. Patients undergoing training with a video game reported significantly higher levels of perceived mental health recovery compared to those in the active control condition (E-book). Patients rated questions related to *Success and Goal Orientation* significantly higher when assigned to the videogame condition compared to the control condition. This maybe a transfer effect since success as well as goal orientation are important elements in the videogame setup being repetitively trained. However, the significant effect on the RAS did not appear immediately but only during follow-up. One reason may be that the RAS measures self-perceived mental health recovery over a certain period of time and those changes, already identified via the PANSS, may not have been consciously accessible to the patient immediately after the intervention but only during follow-up.

Particularly noteworthy is the APVG intervention-related reduction of negative symptomatology which may be due to the rewarding aspects of videogame playing associated with increased activation of dopaminergic neurotransmission; dopamine deficits particularly in prefrontal cortex have been discussed as an underlying pathobiology of negative symptoms in schizophrenia [80]. Further investigations have to clarify possible distinct mechanisms of action.

In summary, the effects of APVG on psychopathology comprise general and negative symptoms measured via the PANSS together with aspects of experienced self-efficacy. This is different to a previous videogame intervention with schizophrenic patients where a beneficial effect on positive symptoms was reported [22]. However, this intervention comprised not one specific videogame but various genres rendering a comparison to our study difficult.

Regardless of the precise training mechanism, our results suggest that training-related beneficial effects may contribute to the treatment of the disease. According to Leucht and colleagues [81] the reported PANSS response rates (75% in the 3D, 70% in the 2D condition relative to only 55% in the active control condition) can be classified as “much improvement” (for a more detailed discussion see supplementary material). Although the respective effect size must be interpreted as rather small, we argue that this effect is clinically meaningful. Especially when considering the advantages of APVG as an adjunctive treatment, including low acquisition cost, ease in use (no major instructions/guidance required) and enjoyable characteristics with might increase the chance that this treatment is applied continuously by the patient. Furthermore, cognitive limitations, negative symptoms and aberrant HC-PFC FC already occur in individuals at high risk [70, 82, 83] and other neurological/psychiatric disorders [84, 85] who may profit from this kind of intervention.

### Limitations

Only 59 out of 69 schizophrenic patients completed the intervention, with a tendency for more dropouts in the APVG intervention conditions resulting in a rather small sample size for this group (for overview see Fig. 1). Although the dropouts in this patient group were expected, they indicate that individuals currently experiencing severe symptoms may not be well-suited for an APVG intervention as cognitive requirements are likely too high. Clinically more stabilised subjects, as well as high-risk candidates, may be more eligible for this kind of intervention. Furthermore, the limited effect sizes within the training period of 8 weeks (ß = 0.09–0.28) for this kind of intervention indicate that APVG training should not be understood as a substitute for relevant pharmacological, social and psychotherapeutic treatments but rather seen as an add-on.

## Conclusion

The present work contributes to disentangling relevant game mechanics assumed to cause the beneficial cognitive effects specifically in sustained attention by linking it to underlying FC change in the HC-PFC network. Importantly, this is the first study providing evidence that playing an APVG reduces global and negative symptoms in patients with schizophrenia, rendering APVGs a possible therapeutic intervention for this and possibly other diseases.

### Supplementary information

Supplement

## Data Availability

The behavioural data and averaged FC values for HC-PFC have been made publicly available at https://github.com/MaxiBecker/SCZ_Training.

## Code availability

The analysis code has been made publicly available at https://github.com/MaxiBecker/SCZ_Training.
