# Supplementary material for: Videogame training increases clinical well-being, attention and hippocampal-prefrontal functional connectivity in patients with schizophrenia
Source: Transl Psychiatry. 2024 May 28;14:218. doi: 10.1038/s41398-024-02945-5 (PMC11133354; doi:10.1038/s41398-024-02945-5)
Supplement: Supplementary file 1 — Supplement [file 41398_2024_2945_MOESM1_ESM.docx]

Videogame training increases clinical well-being, attention and hippocampal-prefrontal functional connectivity in patients with schizophrenia

**Supplementary Material**

**Additional information on the Method section**

*Participants*

The sample size was chosen based on a prior study using the same training intervention [1].

Inclusion Criteria:

- clinically stabilized
- International Classification of Diseases 10 (ICD-10) F20.x (and F25)
- at least one schizophrenic episode in their lives

Exclusion Criteria:

- more than 1h video games per day more than 6 months before study start
- simultaneous major psychiatric disease (if symptomatic in the foreground)
- MRI contraindication
- significant somatic or neurological disease
- significant alcohol or substance abuse in the year before
- clinically relevant anemia
- earlier electroconvulsive shock treatment

*Training procedure*

The story within the two games is similar, aiming to rescue a princess by completing missions. Lost objects must be found and collected in different environments while avoiding enemies and other objects to be able to proceed to higher levels. However, only the 3D condition is a three-dimensional platform game, in which the character needs to explore the virtual environment along the x, y, and z coordinates with a map view for orientation while the 2D version of the game mostly affords a left to right movement along only two dimensions (x and y coordinate). Note, the different game structure resulted in a different average of collected stars in both conditions: 3D:28 stars (SD=30, range=0-126); 2D:5 stars (SD=3, range=0-8).

Importantly, the present study used an active control condition (E-book condition) ruling out placebo effects in the intervention condition to be responsible for the observed improvement.

*Behavioural assessment*

Data acquisition including PANSS ratings, cognitive testing and MR scans were done in two or three sessions each comprising two to four hours. Those sessions took place within an average span of 1.7 days (SD=1.4) in the HP group, and 4.7 days (SD=6.5) in the patient group. Cognitive sessions were not conducted after MR scanning to exclude potential negative effects on concentration.

*Tunnel task.* Participants follow a simulated ride through a tunnel in the first-person perspective comprising straight and curved tunnel segments on a computer screen. The ride through the tunnel can include one to three turns reflecting three difficulty levels. At the tunnel's end, the participants indicate the direction of the start position. After ten test trials, thirty trials were performed where the participants were presented with an arrow after passing the tunnel that had to be positioned in the exact direction of their start position.

This task can be solved by two different strategies: participants with an egocentric frame or *turners* complete this task by changing their orientation as they pass the tunnel; participants with an allocentric frame or *non-turners* remain in their original orientation even after passing the tunnel [2]. Ten categorisation trials, where the participants had to decide between two arrows representing possible solutions (one correct egocentric and one correct allocentric solution), were utilised to assign participants to one of the two navigation strategies. Note however, a considerable part of the participants could not be adequately categorised into one or the other navigation strategy as they continuously switched between both strategies (see Fig. S1). For this reason, we chose to not categorise them a priori to one strategy but decide on a trial by trial basis which strategy they used. Average time on task was 20 minutes.

**
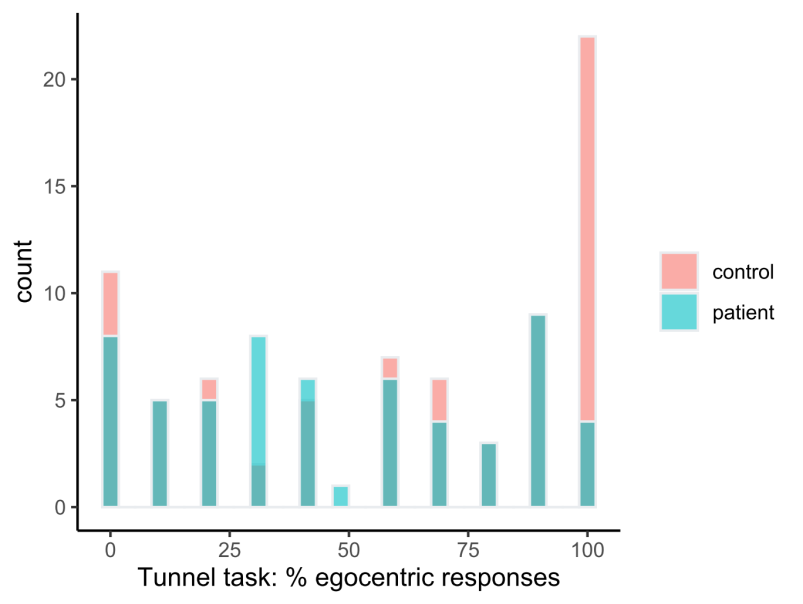
**

**Figure S1.** Proportion of egocentric vs. allocentric responses in Tunnel task during ten categorization trials separately by patients and healthy controls.

*Note.* 0% = 0 egocentric responses; 50% = 5 egocentric responses; 100% = 10 egocentric responses.

*MCCB.* Within this test battery, ten different tasks were performed in a given order (average data acquisition time: 120 minutes). The MCCB captures ten different tasks measuring seven cognitive domains (in that order): speed of processing, working memory, verbal learning, visual learning, reasoning and problem solving, sustained attention and social cognition [3]. Given the focus on neurocognitive performance, the domain of social cognition was not used in the present work and was therefore not included in the overall neurocognitive score of the MCCB. The raw values of each neurocognitive domain were transformed into T-values controlled for age and sex. Green and colleagues [4] tested the different tasks in a sample of N=176 patients with schizophrenia or schizoaffective disorder and used the Intraclass Correlation Coefficient (ICC) to present the Test-Retest Reliability between a baseline measure and a 4-week follow-up ranging from ICC= .68 - .85 (see below).

Processing Speed. Three tasks were applied to assess the processing speed: Trail Making Test, Brief Assessment of Cognition in Schizophrenia: Symbol Coding as well as Category Fluency: Animal Naming. In the Trail Making Test, the participants were instructed to connect randomly arranged numbers from one to twenty-five in the correct order as quickly as possible. The participants' challenge in the Brief Assessment of Cognition in Schizophrenia was to match as many randomly displayed symbols to the corresponding numbers (indicated on the solution key) as quickly and correctly as possible within a limited time of 90 seconds. In addition, a test of verbal processing speed was added in the form of the Category Fluency task [3]. In this test, the participants were instructed to name as many animals as possible within a time of 60 seconds. Trail Making Test: ICC=0.75; Brief Assessment of Cognition in Schizophrenia: ICC=0.85; Category Fluency: ICC=0.74*.*

Verbal learning. The Hopkins Verbal Learning Test-Revised was used to assess verbal learning by asking the participants to immediately repeat a list of twelve unsorted words from 3 semantic categories. ICC=0.68.

Visual learning. A paper with six different geometric shapes was presented to the participants for exactly ten seconds in the Brief Visuospatial Memory Test-Revised. The participants were then asked to reproduce the geometric shapes as accurately as possible on a blank sheet. This test procedure was repeated twice. ICC=0.71.

Working Memory. Two tasks were used to measure the working memory performance: Wechsler Memory Scale: Spatial Span and the Letter-Number Span. The Wechsler Memory Scale measures the ability to remember the correct sequence of consecutive blocks. In both sections (forward and backwards order) the WMS-Spatial-Span board was used, on which ten blue blocks are placed. After the instructor touched the blocks in a certain order, the participants were asked to repeat them (depending on the section) in forwards or backwards order. In the Letter-Number Span, the participants were presented with different sequences consisting of disordered letters and numbers. These had to be ordered by the participants as follows: first, the numbers are named from the smallest to the largest and second the letters are named in alphabetical order. Wechsler Memory Scale: ICC=0.74; Letter-Number Span: ICC=0.78.

Reasoning & Problem solving. To assess performance in reasoning and problem solving the Neuropsychological Assessment Battery: Mazes-Test was used. The participants were instructed to solve six different maze tasks with an increasing level of difficulty as quickly and correctly as possible. ICC=0.83.

Sustained Attention. In the Continuous Performance Test, the attention vigilance was measured by a computer-based test, presenting visual white numbers on a black computer screen. The participants had to respond as quickly and accurately as possible when two identical numbers were presented successively. The task began with a block of two-digit numbers and increased up to the block with four-digit numbers. A sensitivity index [D'Prime] was given for each of the three sequences as a measure of the sensitivity achieved to discriminate the target stimuli from the wrong stimuli. ICC=0.84.

*Clinical assessment*

*PANSS.* The Positive and Negative Syndrome Scale evaluates the symptom severity of the past seven days on a seven-point Likert scale (higher values indicating stronger severity). The 30 items were grouped into three subscales: a Positive scale (seven items, including e. g. symptoms of delusions and suspiciousness), a Negative scale (seven items, including e. g. symptoms of blunted affect and poor rapport), and a General Psychopathology scale (sixteen items, including e. g. anxiety and disorganisation) [5]. The PANSS ratings were carried out by two (out of four) clinically trained individuals, whose ratings were averaged for the final score (interrater reliability: r_s_ = .97). The raters had no knowledge about the patient’s respective condition (E-book, 2D or 3D videogame) and one rater always remained constant throughout the four assessments per patient. For a more detailed picture of the symptom development, the PANSS was additionally assessed after the fourth week of the intervention.

Interrater concordance using Pearson’s correlation is *r*=.83 for the positive syndrome subscale, r=.85 for the negative syndrome subscale and r = .87 for the GPS among three assessors [5].

*RAS.* In the German version of the recovery assessment scale (RAS), the participants were instructed to rate 24-items on a 5-point Likert scale (ranging from 1 ‘‘strongly disagree’’ to 5 ‘‘strongly agree’’). Five sub-factors based on Corrigan and colleagues [6] and a total score were computed, with higher scores indicating higher perceived mental health recovery. The sub-factors are Personal confidence and hope (e. g. “I can handle what happens in my life”), Willingness to ask for help (e. g. “I ask for help when I need it”), Goal and success orientation (e. g. “I have goals in life that I want to reach”), Reliance on others (e. g. “I have people I can count on”), No dominated by symptoms (e. g. “My symptoms interfere less and less with my life”)*.*

In a sample of *N* = 156 participants diagnosed with either schizophrenia or schizoaffective disorder the German version of the RAS resulted in Cronbach’s alpha ranging from α=0.88 for the subscale willingness to ask for help to α=0.60 for the subscales no dominated by symptoms and reliance on others [7].

*Behavioural data analysis*

All analyses were carried out in R (version 4.3.0, R Core Team, 2014) using the lme4-package (version 1.1-33, Bates et al., 2014). P-values for the condition*time interaction are reported from the exact Chi² test-statistic of the likelihood-ratio tests comparing the fit of the model with and without the interaction term (note, we only report the best-fitting model according to the exact Chi² test-statistic). P-values for all single predictors from those models were obtained via t-tests from the lmerTest-toolbox (version 3.1-3) using the Satterthwaite’s method for denominator degrees of freedom [8]. Standardized beta coefficients and their 95% confidence intervals were estimated via the effectsize-Toolbox (version 0.8.6) in R.

*Covariates.* Kühn and colleagues [1] found that one factor explaining the effects of gaming on cognition and brain was the perceived fun and the experienced pleasure that playing a videogame invokes. To investigate the experienced pleasure while dealing with the respective media device and control for potential differences between the conditions, we conducted a weekly questionnaire comprising five questions measuring the extent of experienced pleasure while playing or reading in all three conditions (see below). The response of all five questions for all eight weeks was averaged. Missing values were replaced by respective condition means (E-book=4.6, *SD*=1.2; 2D=4.28, *SD*=0.91; 3D: *M*=3.8, *SD*=1.04). In fact, the amount of experienced pleasure (henceforth *fun* variable) differed between the groups as the 3D condition experienced significantly less fun compared to the 2D (*t*(127)=-2.13, *p*<.05) and E-book condition (*t*(127)=-3.44, *p*<.001). We, therefore, entered *fun* as a covariate into all models. For clinical assessments (PANSS, RAS), we further added a variable describing an Olanzapine equivalence doses (henceforth *medication* variable) as covariate of no interest to minimise the effect of antipsychotic medication on those variables [9]. For the cognitive tasks (MCCB, Tunnel task) which were performed by both patients and HP, we included both groups in the same model to maximally inform the model when estimating those outcome variables. To investigate whether patients performed differently from HP in the respective condition over time, we additionally calculated a three-way interaction with condition*time*group. The group variable was dummy coded differentiating between patients and HP. Note, due to collinearity we did not include the medication and group variable in the same model (τ=.74, p<.001). For the Tunnel task we additionally modelled the amount of turns reflecting the level of difficulty (1-3, ordered variable, henceforth *difficulty;* see Table 2).

*Weekly questionnaire (translated to English from German) comprising the* ***fun*** *variable*

Question 1) I feel irritated.

Question 2) I wish I could have read / played the book / game longer.

Question 3) I feel great after playing the game / reading the book.

Question 4) I enjoy playing the game / reading the book

Question 5) I found it very enjoyable to read the book / play the game.

When modelling the subscores for the MCCB and RAS, p-values from the likelihood ratio tests were corrected for multiple comparison using Bonferroni correction [10] for those subscores where we did not have an a priori hypothesis (as for the CPT from the MCCB measuring sustained attention). All dependent variables were modelled assuming a Gaussian error distribution with the default identity link function. The dependent variable of the Tunnel task was log transformed to reach normality. Visual inspection of the residual plots for all models did not reveal obvious deviations from normality and homoscedasticity. Note, degrees of freedom reported for predictors estimated via the lmer-Test function were estimated using the Satterthwaite approximation method [8].

*MRI data analysis*

*Preprocessing & Denoising.* Functional scans were first realigned: all scans were co-registered and resampled to the first reference image using b-spline interpolation [11]. Functional scans were then slice-time corrected and outlier images were identified using framewise displacement and CONN’s default parameters. After normalising functional and structural data into MNI space they were segmented into grey, white matter as well as CSF tissue. Finally, the functional data was smoothed using an 8mm full width half maximum Gaussian kernel to increase BOLD signal to noise-ratio.

CONN’s implemented anatomical component-based noise correction procedure (CompCor) was used to remove confounding effects to the estimated BOLD signal for each voxel in each subject and each time point, i.e. session. Those potential confounding effects comprise six subject-motion parameters including their first-order derivatives, constant or linear condition effects as well as identified outlier scans and noise components from areas of cerebrospinal fluid and cerebral white matter. After visual inspection of the denoising effects using CONN’s quality control panels, the resulting time series were band-pass filtered to .008 - .09 Hz.

*First & Second Level Analysis*. CONN’s default network atlas representing ROIs from commonly networks based on ICA analyses of the Human Connectome Project (497 subject; Whitfield-Gabrieli and Nieto-Castanon [12]) comprises the following prefrontal ROIs: bilateral IFG (language network), bilateral rostral prefrontal cortex (RPFC, salience network), bilateral anterior insula (AI, salience network), anterior cingulate cortex (ACC, salience network), medial prefrontal cortex (MPFC, default mode network), bilateral lateral prefrontal cortex (LPFC, central executive network) and bilateral frontal eyefields (FEF, dorsal attention network).

Each element in each connectivity matrix represents the Fisher-transformed bivariate correlation coefficient between the preprocessed and denoised BOLD time series of the two ROIs.

To investigate the brain-behaviour link, we first normalized and mean centered the behavioural difference values (post-pre) before entering them into CONN. Subsequently, we estimated the effect of those variables on all HC-PFC connections in the network controlling for the effect of the group (patient or HP) and the fun variable (between subject contrast: 1*variable + 0*fun + 0*group; within subject contrast: -1*pre + 1*post intervention). Note, we added the *medication* variable (Olanzapine equivalent doses, see above) as covariate of no interest, when modelling only variables including patients (i.e., PANSS, RAS) that did not include the group variable since medication and group were highly collinear. The resulting p-values were FDR-corrected for the amount of existing PFC-ROIs in the HC-PFC network.

**Additional information of the results section**

*
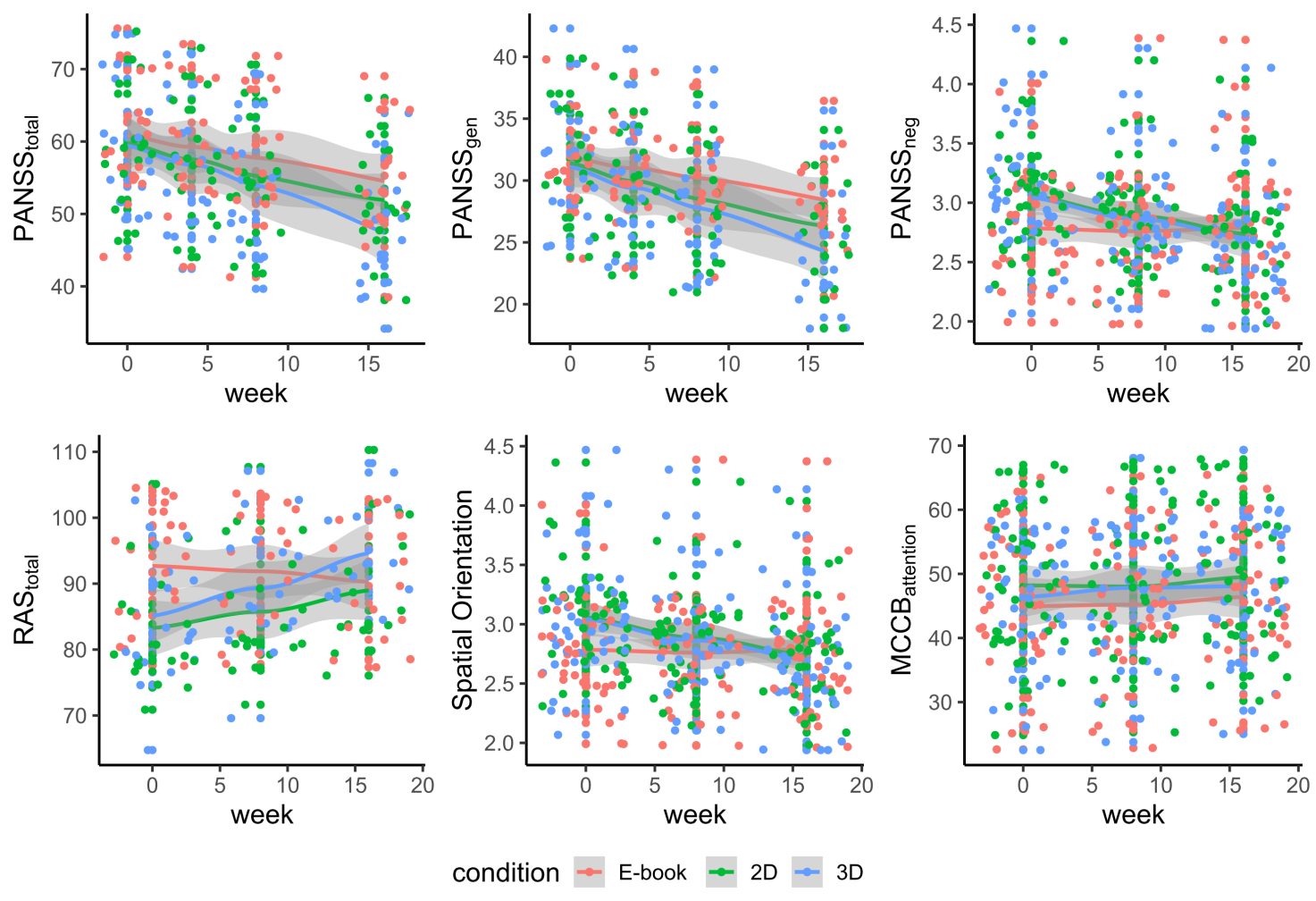
*

***Figure S2.*** Effects of training intervention on cognition - spatial orientation and sustained attention and clinical well-being - PANSS and RAS over the time course of 16 weeks.

*Note*. PANSS_gen_ = General Psychopathology Subscale, PANSS_neg_ = Negative Subscale, values represent estimated marginal means. Coloured shades around regression lines represent 95% between-subject confidence intervals. *Spatial Orientation* shows the angle deviance between the estimated and correct direction, with lower values indicating better performance.

**Longitudinal Impact of Training Intervention on Cognition and Symptomatology across 16 Weeks**

To assess whether the identified linear trends in cognition (particularly in sustained attention and, to a lesser extent, spatial orientation) and symptomatology (measured through PANSS) persisted beyond the initial 8-week active training period, we conducted an additional exploratory analysis. This involved re-analyzing the same models described in the main text for the initial 8-week timeframe, but with the inclusion of corresponding values at the follow-up assessment eight weeks after training completion.

Cognition. A significant linear trend was observed for the Tunneltask (spatial orientation, *t*(978.4)=--2.95, *p*=.003, *ß*=-.05, *CI*[-.03 -.08]) after 16 weeks. Posthoc analyses revealed that both the 3D (*t*(978.4)=-2.95, *p*=.003, *ß*=-.08, CI[-.04 -.12]) and 2D (*t*(966.7)=-2.92, *p*=.003, *ß*=-.07, *CI*[-.03 -.11]) conditions performed significantly better (i.e. smaller angle deviance) than the Kindle control condition, with no significant difference between the 3D and 2D condition (*p*>.95). No significant linear trend for the condition*time interaction was found for the CPT (sustained attention; p>.76).

PANSS. No significant negative linear trend was observed anymore for the condition*time interaction in the PANSS_total_ (*t*(172.47)=-1.765, *p*=.079, *ß*=-.12, *CI*[.01 -.26]) or PANSS_neg_ (*t*(172.5)=-1.405, *p*=.162, *ß*=-.10, *CI*[.04 -.24]). However, a negative linear trend persisted in PANSS_gen_ over the 16-week period (*t*(172.4)=-2.07, p=.040, *ß*=-.14, *CI*[-.01 -.28]). Posthoc analysis indicated a stronger reduction in symptom expression in the 3D condition compared to the Kindle control condition (*t*(172.3)=-2.07, *p*=.040, *ß*=-.20, *CI*[-.01 -.40]), with no significant differences observed between the 3D and 2D condition (p>.62), or the 2D and Kindle condition (p>.11).

| **Table S1.** | | | | |  | |  | | | |  | | |  | | |  | | | |  | | |
| --- | --- | --- | --- | --- | --- | --- | --- | --- | --- | --- | --- | --- | --- | --- | --- | --- | --- | --- | --- | --- | --- | --- | --- |
| Results of Mixed Model: Predicting sustained attention and spatial orientation as a function of training intervention | | | | | | | | | | | | | | | | | | | | | | | |
|  | | | | | **MCCB: Attention** | | | | | | | | | **Tunnel: Spatial orientation** | | | | | | | | | |
| Predictors | | | | | ß | | CI | | | | p | | | ß | | | CI | | | | p | | |
| \| (Intercept) \| 0.40 \| 0.21 – 0.60 \| **<0.001** \| 0.57 \| 0.50 – 0.64 \| **<0.001** \| \| --- \| --- \| --- \| --- \| --- \| --- \| --- \| \| condition [l] \| 0.32 \| 0.06 – 0.58 \| **0.017** \| 0.05 \| -0.05 – 0.15 \| 0.297 \| \| condition [q] \| -0.09 \| -0.35 – 0.16 \| 0.466 \| -0.02 \| -0.11 – 0.08 \| 0.695 \| \| time \| 0.04 \| -0.01 – 0.09 \| 0.083 \| -0.03 \| -0.05 – 0.15 \| **0.008** \| \| group [patient] \| -0.87 \| -1.16 – -0.58 \| **<0.001** \| 0.07 \| -0.04 – 0.17 \| 0.238 \| \| fun \| 0.13 \| -0.03 – 0.28 \| 0.103 \| 0.00 \| -0.06 – 0.06 \| 0.951 \| \| condition [l] * time \| 0.08 \| 0.00 – 0.17 \| **0.046** \| -0.05 \| -0.08 – -0.02 \| **0.002** \| \| condition [q] * time \| -0.08 \| -0.17 – 0.00 \| 0.054 \| 0.01 \| -0.03 – 0.04 \| 0.698 \| \| difficulty[l] \|  \|  \|  \| 0.12 \| 0.09 – 0.15 \| **<0.001** \| \| difficulty[q] \|  \|  \|  \| 0.07 \| 0.03 – 0.10 \| **<0.001** \| \| **Random Effects** \|  \|  \|  \|  \|  \|  \| \| σ2 \| 20.76 \| \| \| 0.30 \| \| \| \| ICC \| 0.81 \| \| \| 0.48 \| \| \| \| N \| 134 \| \| \| 135 \| \| \| \| Marg. R² / Cond. R² \| 0.220 / 0.849 \| \| \| 0.075 / 0.521 \| \| \|   *Note.* [l] = linear; [q] = quadratic; ß = standardised Betas, CI = 95% confidence interval, ICC = intraclass correlation coefficient, N= amount of subjects in analysis, Condition = 3D>2D>E-book; time= 1st - 8th week. P-values for individual predictors are obtained via t-tests using Satterthwaite’s method for denominator degrees of freedom (from the lmerTest-toolbox). | | | | | | | | | | | | | | | | | | | | | | | |
| **Table S2.** | | | | | | | | | | | | | | | | | | | | | | | |
| Results of Mixed Model - Predicting clinical symptoms as a function of the training intervention | | | | | | | | | | | | | | | | | | | | | | | |
|  | **PANSS_total_** | | | | | | | **PANSS_gen_** | | | | | | | | | **PANSS_neg_** | | | | | | |
| Predictors | ß | | CI | | | | p | ß | | | CI | | | | p | | ß | | CI | | | p | |
| (Intercept) | 0.01 | | -0.20 – 0.22 | | | | 0.958 | 0.01 | | | -0.20 – 0.22 | | | | 0.944 | | -0.01 | | -0.23 – 0.21 | | | 0.962 | |
| condition [l] | -0.17 | | -0.54 – 0.20 | | | | 0.371 | -0.20 | | | -0.58 – 0.17 | | | | 0.284 | | -0.03 | | -0.42 – 0.36 | | | 0.877 | |
| condition [q] | 0.06 | | -0.31 – 0.42 | | | | 0.753 | 0.06 | | | -0.30 – 0.43 | | | | 0.726 | | -0.02 | | -0.41 – 0.36 | | | 0.900 | |
| time | -0.25 | | -0.34 – -0.17 | | | | **<.001** | -0.25 | | | -0.33 – -0.16 | | | | **<.001** | | -0.06 | | -0.15 – 0.02 | | | 0.151 | |
| fun | 0.03 | | -0.19 – 0.24 | | | | 0.802 | -0.04 | | | -0.26 – 0.18 | | | | 0.720 | | 0.04 | | -0.19 – 0.27 | | | 0.735 | |
| medication | 0.12 | | -0.06 – 0.29 | | | | 0.185 | 0.10 | | | -0.07 – 0.27 | | | | 0.258 | | 0.04 | | -0.14 – 0.22 | | | 0.644 | |
| condition [l]* time | -0.16 | | -0.31 – -0.01 | | | | **0.036** | -0.16 | | | -0.31 – -0.01 | | | | **0.038** | | -0.16 | | -0.31 – -0.01 | | | **0.040** | |
| condition[q]* time | 0.15 | | -0.00 – 0.30 | | | | 0.051 | 0.19 | | | 0.04 – 0.34 | | | | **0.012** | | 0.14 | | -0.01 – 0.29 | | | 0.077 | |
| **Random Effects** | | | | | | |  |  | | |  | | | |  | |  | |  | | |  | |
| σ^2^ | 37.74 | | | | | | | 12.63 | | | | | | | | | 4.81 | | | | | | |
| ICC | 0.63 | | | | | | | 0.63 | | | | | | | | | 0.65 | | | | | | |
| N | 59 | | | | | | | 59 | | | | | | | | | 59 | | | | | | |
| Marg.R^2^ / Cond. R^2^ | 0.107 / 0.674 | | | | | | | 0.109 / 0.670 | | | | | | | | | 0.022 / 0.660 | | | | | | |
| *Note.* Gen = General Psychopathology subscore; Neg = Negative subscore; [l] = linear; [q] = quadratic; ß = standardized Betas, CI = 95% confidence interval, ICC = intraclass correlation coefficient, N= amount of subjects in analysis, Condition = 3D>2D>E-book; time= pre vs post intervention; medication = Olanzapine equivalence doses of antipsychotic medication; P-values for individual predictors are obtained via t-tests using Satterthwaite’s method for denominator degrees of freedom (from the lmerTest-toolbox). | | | | | | | | | | | | | | | | | | | | | | | |
| **Table S3.** | | |  | | |  | | | |  | | |  | |  | | | |  | | |  |  |
| Results of Mixed Model: Predicting clinical recovery (RAS) as a function of training intervention | | | | | | | | | | | | | | | | | | | | | |  |  |
|  | | | **RAS_total_** | | | | | | | | | | **RAS_Success & Goal Orientation_** | | | | | | | | |  |  |
| Predictors | | | ß | | | CI | | | | p | | | ß | | CI | | | | p | | |  |  |
| \| (Intercept) \| 0.00 \| -0.18 - 0.19 \| 0.968 \| 0.01 \| -0.19 – 0.20 \| 0.944 \| \| --- \| --- \| --- \| --- \| --- \| --- \| --- \| \| condition [l] \| 0.01 \| -0.33 – 0.34 \| 0.965 \| -0.07 \| -0.42 – 0.27 \| 0.676 \| \| condition [q] \| 0.38 \| 0.05 – 0.70 \| **0.024** \| 0.15 \| -0.18 – 0.48 \| 0.374 \| \| time \| 0.14 \| 0.05 – 0.23 \| **0.002** \| 0.11 \| 0.01 – 0.21 \| **0.031** \| \| fun \| 0.43 \| 0.23 – 0.63 \| **<0.001** \| 0.38 \| 0.18 – 0.58 \| **<0.001** \| \| medication \| -0.05 \| -0.21 – 0.11 \| 0.547 \| -0.07 \| -0.24 – 0.09 \| 0.396 \| \| condition[l]*time \| 0.26 \| 0.10 – 0.42 \| **0.001** \| 0.28 \| 0.12 – 0.46 \| **0.001** \| \| condition [q]*time \| -0.03 \| -0.19 – 0.11 \| 0.690 \| 0.03 \| -0.13 – 0.20 \| 0.742 \| \| **Random Effects** \|  \|  \|  \|  \|  \|  \| \| σ² \| 51.93 \| \| \| 0.15 \| \| \| \| ICC \| 0.56 \| \| \| 0.52 \| \| \| \| N \| 59 \| \| \| 59 \| \| \| \| Marg. R² / Cond. R² \| 0.255 / 0.670 \| \| \| 0.192 / 0.616 \| \| \|   *Note*. RAS = recovery assessment scale; [l] = linear; [q] = quadratic; ß = standardised Betas, CI = 95% confidence interval, ICC = intraclass correlation coefficient, N= amount of subjects in analysis, Condition = 3D>2D>E-book; time=1st -16th week; medication = Olanzapine equivalence doses of antipsychotic medication; Cond.R² = Conditional R². P-values for individual predictors are obtained via t-tests using Satterthwaite’s method for denominator degrees of freedom (from the lmerTest-toolbox). | | | | | | | | | | | | | | | | | | | | | |  |  |

**Response rates and patient characteristics predicting treatment response**

To further explore the amount of patients that responded to the APVG treatment, we calculated response rates for each outcome variable (sustained attention, spatial orientation, PANSS_total_, RAS_total_). Response rates were calculated as the percentage of change in performance from the pre to the post intervention timepoint (note, for RAS we used the follow-up instead of the post intervention timepoint and we only saw a treatment response at follow-up) relative to the pre intervention timepoint (see Table S4).

For a better understanding of the clinical meaning, the PANSS response rates were converted into the Clinical Global Impressions Scale[13] (CGI). This procedure, described by Leucht and colleagues[14], enables the categorisation of the percentage change in the PANSS to be described with the extent of CGI improvement scale. Using a seven-point Likert scale (1=very much improved, 2=much improved, 3=slightly improved, 4=no change, 5=slightly worse, 6=much worse, 7=very much worse), this scale evaluates the patient's improvement or worsening compared to a baseline. Leucht and colleagues[14] suggests that a minimum 50% reduction in PANSS scores can be considered a significant improvement, falling under the category of "much improved".

Furthermore, we explored which patient characteristics predicted treatment response. To achieve this, we constructed four multiple regression models, each tailored to predict one of the outcome variables (MCCB: Sustained Attention, Tunnel: Spatial Orientation, PANSS_total_, RAS_total_) reflecting treatment response assessed after the eighth week (or after the 16^th^ week for RAS_total_, respectively). Those regression models included the following independent variables as patient characteristics: *age*, *sex*, *year of education*, change in *Olanzapine equivalent doses* (difference between post and pre timepoints or difference between follow-up and pre timepoints for the RAS_total_ respectively), *number of psychotic episodes*, and *onset age of first psychotic episode*. Treatment response in % for all four variables was quantified as described further above. No signs of multicollinearity were found (VIF<2.47) for any of the regression models.

Onset age of the first psychotic episode was found to have a negative association with treatment response in sustained attention adjusted for age (*t*(49)=-2.20, *p*=.032, ß=-.40, CI[-.76,-.04]). This suggests that individuals who experienced their first psychotic episode earlier tended to exhibit a stronger response to attention-related treatment, regardless of their current age. No other patient characteristic emerged as a predictor of sustained attention. None of the other patient characteristics predicted treatment response in RAS_total_, spatial orientation measured via the tunnel task, or symptomatology measured via the PANSS_total_.

| **Table S4** | | |  | | | | |  | | | |  | | | |  | |  | |
| --- | --- | --- | --- | --- | --- | --- | --- | --- | --- | --- | --- | --- | --- | --- | --- | --- | --- | --- | --- |
| Response rates (in %) in patient sample on behavioral outcome variables divided by quantiles | | | | | | | | | | | | | | | | | | | |
|  | | | no Response | | | | | Response | | | | | | | | | | | |
|  | | |  |  |  |  |  | 1<>25 % | | | | 25<>50 % | | | | 50<>75 % | | 75<>100 % | |
| Group | | |  | | | | |  | | | |  | | | |  | |  | |
| **MCCB: Attention** | | | | | | | | | | | | | | | | | | | |
| 3D game | | | 40% | | | | | 10% | | | | 5% | | | | 0% | | 45% | |
| 2D game | | | 45% | | | | | 5% | | | | 10% | | | | 5% | | 35% | |
| E-book | | | 58% | | | | | 5% | | | | 16% | | | | 5% | | 16% | |
|  | | |  | | | | |  | | | |  | | | |  | |  | |
| **Tunnel: Spatial Orientation** | | | | | | | | | | | | | | | | | | | |
| 3D game | | | 22% | | | | | 6% | | | | 0% | | | | 0% | | 72% | |
| 2D game | | | 47% | | | | | 5% | | | | 16% | | | | 5% | | 26% | |
| E-book | | | 45% | | | | | 0% | | | | 0% | | | | 0% | | 55% | |
|  | | |  | | | | |  | | | |  | | | |  | |  | |
| **PANSS_total_** | | | | | | | | | | | | | | | | | | | |
| 3D game | | | 15% | | | | | 5% | | | | 5% | | | | 0% | | 75% | |
| 2D game | | | 15% | | | | | 10% | | | | 5% | | | | 5% | | 65% | |
| E-book | | | 40% | | | | | 0% | | | | 5% | | | | 5% | | 50% | |
|  | | |  | | | | |  | | | |  | | | |  | |  | |
| **RAS_total_** | | | | | | | | | | | | | | | | | | | |
| 3D game | | | 25% | | | | | 6% | | | | 13% | | | | 25% | | 31% | |
| 2D game | | | 41% | | | | | 18% | | | | 6% | | | | 12% | | 24% | |
| E-book | | | 59% | | | | | 12% | | | | 6% | | | | 24% | | 0% | |
| *Note*. PANSS = Positive and Negative Syndrome Scale; RAS = Recovery Assessment Scale. *No response* values represent the amount of subjects in % with no or performance decrease in each group. Performance increase (i.e. treatment response) for each outcome variable is divided into quantiles and presented individually for each group. | | | | | | | | | | | | | | | | | | | |
| **Table S5.** | | | | | | | | | | | | | | | | | | |  |
| Individual connections in HC-PFC FC network exhibiting condition * time interaction | | | | | | | | | | | | | | | | | | |  |
| 1st ROI | | | | | | | 2nd ROI | | | | | | | | Statistic | | | |  |
| ROI | H | x | | y | z | NW | ROI | | H | x | y | | z | NW | T (df=127) | | p-unc | |  |
| HC | R | 0 | | 0 | 0 | 0 | RPFC | | L | -32 | 45 | | 27 | SAL | 3.10 | | .001 | |  |
| HC | R | 0 | | 0 | 0 | 0 | RPFC | | R | 32 | 46 | | 27 | SAL | 1.92 | | .028 | |  |
| HC | L | 0 | | 0 | 0 | 0 | RPFC | | L | -32 | 45 | | 27 | SAL | 2.65 | | .004 | |  |
| HC | L | 0 | | 0 | 0 | 0 | RPFC | | R | 32 | 46 | | 27 | SAL | 2.44 | | .008 | |  |
| HC | L | 0 | | 0 | 0 | 0 | ACC | | LR | 0 | 22 | | 35 | SAL | 2.45 | | .008 | |  |
| MPFC | LR | 1 | | 55 | -3 | DMN | RPFC | | L | -32 | 45 | | 27 | SAL | 2.00 | | .023 | |  |
| LPFC | L | -43 | | 33 | 28 | FP | RPFC | | L | -32 | 45 | | 27 | SAL | 2.02 | | .022 | |  |
| FEF | L | -27 | | -9 | 64 | DA | RPFC | | R | 32 | 46 | | 27 | SAL | 2.64 | | .004 | |  |
| *Note*. H=hemisphere; RPFC = Rostral PFC; ACC = Anterior Cingulate Cortex; HC = Hippocampus; FEF = Frontal Eye Fields, LPFC = lateral prefrontal cortex; MPFC = medial prefrontal cortex; NW = network; Sal = Salience; DA = dorsal attention; FP = frontoparietal; DMN = default mode; N=132. | | | | | | | | | | | | | | | | | | |  |

**Data report of other preregistered training-related outcome measures**

**Magnetic Resonance Spectroscopy (MRS) – Glutamate**

Current pathophysiological theories of schizophrenia highlight the role of glutamate in altered brain connectivity [15]. Reciprocal connections between corticocortical and corticolimbic including hippocampal projections are exclusively glutamatergic. Glutamatergic neurons are, therefore, the exclusive means by which (aberrant) information is transferred within and between these regions [16]. Furthermore, glutamate modulates long-term potentiation and thereby plays an essential role in memory and learning processes. For those reasons, we explored the neurochemical basis of FC in relation to the videogame intervention. We hypothesized that changes in absolute glutamate concentration may predict training-related changes in HC-PFC FC.

Method. We chose to measure absolute glutamate concentrations in a 20 × 20 × 12mm voxel in the right posterior HC. MRS data were acquired with 3-Tesla 1H-MRS using water-suppressed and unsuppressed spectra [point resolved spectroscopy; 128/8 averages; 90° flip angle; repetition time (TR), 3 s; echo time (TE), 80 ms; automatic shimming] and they were collected in the same session after acquisition of resting state fMRI. MRS data were analyzed using the commercial spectral-fitting package Linear Combination of Model spectra (LCModel) [17], including water-suppressed and unsuppressed spectra. All MRS voxels were individually placed by anatomically trained MR operators. Due to lower MRS signal-to-noise ratio in deep brain structures [18], glutamate in the HC was measured using a more liberal definition of Cramér–Rao lower bounds of <40% fit deviation (mean fit deviation, 20.4%; SD, 7.25%; range, 1–40%). Because glutamate measured via MRS represents the total content of glutamate in respective MRS voxel [19] independent of brain tissue compartments, this voxel was additionally segmented in GM, white matter (WM), and CSF fractions using the unified segmentation approach [20] in Python software (Python Software Foundation) based on the T1 structural image. Absolute glutamate concentrations were subsequently adjusted for WM and GM content within the voxel using the formula: adjusted glutamate = absolute glutamate * [1/(GM + WM)]. We report only adjusted glutamate concentrations with the unit millimoles per litre. Glutamate concentration was analysed in the same way as the MCCB behavioural data using a linear mixed model with the same independent variables. Extreme values higher than three SD from the mean were excluded and glutamate concentration was further log-transformed to reach normality. Furthermore, we tested whether changes in adjusted glutamate concentration (difference value: post-pre intervention) correlated with training-related changes in HC-PFC functional connectivity and for exploratory purposes attention (difference value: post-pre intervention) independent of the conditions including group and fun as covariate. Because the difference value for glutamate was not normally distributed, we first regressed out group and fun and subsequently performed a Spearman’s rank order correlation on the residuals with HC-PFC functional connectivity and attention.

Results/Discussion. We did not find evidence for a condition*time effect on glutamate concentration (Chi²(2)=0.11, p=.944). No other predictor was significant (p>.31). Furthermore, we found no evidence for a correlation between adjusted glutamate concentration in the right HC and HC-PFC FC (r_s_=-.12, p=.22) nor sustained attention (r_s_=-.13, p=.19). Note, however, because a voxel was chosen in right HC - a rather deep brain structure - data quality obtained from this voxel was rather low as indicated by high fit deviation = 20.4%. As data >40% fit deviation was excluded this resulted in high data loss (N=99). This in combination with a smaller sample size specifically for patients may be the reason why we did not find a condition*time effect in glutamate concentration as power may have been too low to detect a significant difference. Future studies should therefore investigate training related changes between glutamate and HC-PFC FC as well as cognition using a bigger sample size and measuring glutamate concentration not only in HC but also in prefrontal regions as the signal to noise ratio is higher.

**T1 MRI - brain volume**

Despite evidence for a training related change in functional connectivity, evidence also points to an increase in structural plasticity specifically in HC and PFC as a function of playing action platform videogames [1, 21]. For this reason, we additionally assumed a training related increase in grey matter volume specifically in PFC and HC.

Method. Structural images were obtained as described above using a three-dimensional T1-weighted magnetization prepared gradient-echo sequence (MPRAGE) (repetition time = 2500 ms; echo time = 2.12ms; TI = 1100 ms, acquisition matrix = 240 × 241 × 194, flip angle = 9˚; 0.8 x 0.8 x 0.94 mm voxel size). We obtained grey matter volume estimates using CAT12 (v1278) running on SPM12 and Matlab R2016b using default parameters. The longitudinal processing pipeline was chosen using default parameters according to the standard protocol (<http://www.neuro.uni-jena.de/cat12/CAT12-Manual.pdf>). CAT12 automatically performs intra-subject realignment, bias correction, segmentation, and normalization. Segmentation and normalization were realized with default parameters segmenting the structural images into three voxel classes (grey matter [GM], cerebrospinal fluid [CSF] and white matter [WM]) using adaptive maximum a posteriori segmentation and partial volume segmentation. To increase signal to noise ratio, the extracted GM maps were subsequently smoothed with an 8mm FWHM kernel. Quality control was executed during two stages: First, images were visually inspected for artefacts prior to automatic processing. Second, the images were manually checked for inter-subject homogeneity after segmentation using the *check homogeneity* function in CAT12. After preprocessing, the images were manually checked for data quality. Due to our specific hypothesis, we used a region-of-interest (ROI) based approach making use of the SPM’s neuromorphometrics atlas to segment grey matter brain volume in HC and PFC ROIs (Neuromorphometrics, Inc. (http://Neuromorphometrics.com/). We chose all available PFC ROIs in this atlas and analysed the data using mixed models using the following formula: ROI ~ condition * time * group + fun + GM + (1|subjects) + ε. ROI represents the grey matter volume of the respective HC or PFC ROI and we included the subject’s overall grey matter volume (GM) as additional covariate of no interest. Extreme values higher than three SD from the mean were excluded.

Results/Discussion. The results are presented in Table S6 with uncorrected p-values and separately for each hemisphere. Patients with schizophrenia exhibited less brain volume in the right HC, right Inferior Orbital Frontal Gyrus, bilateral Middle Cingulate Gyrus, right Frontal Pole as well as right Middle Frontal Gyrus compared to healthy controls (FDR-corrected). However, despite prior evidence using the same (3D) videogame (Kühn, Gleich [1] and replicated by West, Zendel [21]), we found no evidence for a training related increase in HC or PFC grey matter volume neither in healthy controls nor in patients with schizophrenia (p-uncorr>.29). This was unexpected. A possible reason for this lack of evidence could be the heterogeneity of our sample. While mostly female (M=73%), young (M=24.1; SD=3.8) (medical) students were recruited in Kühn and colleagues [1], the present study includes healthy participants that are not only generally older (M=29.92) but also more diverse in age (SD=8.25, min= 18 years, max= 54 years) and their level of education (mostly non-students; educational years: M=16.43; SD=3.49) as well as the amount of included females (M=43%) was lower because they were matched to the schizophrenic patients. Finally, the observed null-effects may also be due to the choice of the control condition: The present study used an active instead of a passive control condition (reading a Kindle). Both Kühn and colleagues [1] as well as West and colleagues [21] found significant effects only between the videogaming and a passive control condition. However, West and colleagues [21] found no difference between the videogaming and an active control condition (taking a series of self-directed, computerized piano lessons). Hence, even though a recent metaanalysis showed no meaningful performance differences between active and passive control conditions in cognitive interventions for healthy adults (d=.027, Au, Gibson [22]), it is currently unclear whether this is also the case for structural brain effects in videogaming interventions. As a consequence, future videogame training studies should more systematically investigate effect size differences between active and passive control conditions in such interventions.

| **Table S6.** | | | | | | | | | | |
| --- | --- | --- | --- | --- | --- | --- | --- | --- | --- | --- |
| HC PFC gray matter volume as function of videogame intervention | | | | | | | | | |  |
|  | H | GM | Cond. [l] | Cond. [q] | time | group [pat.] | fun | Cond. [l] * time | Cond. [q] * time | Marg. R^2^ / Cond. R^2^ |
| **HC** | l | 0.13 ^***^ | -0.01 | -0.02 | -0.00 | -0.02 | 0.00 | 0.00 | -0.00 | 0.402 / 0.652 |
|  | r | 0.17 ^***^ | -0.01 | -0.01 | -0.00 | -0.09 ^**^ | 0.02 | 0.01 | -0.01 | 0.449 / 0.659 |
| **ParaHC** | l | 0.17 ^***^ | 0.05 ^*^ | -0.01 | 0.00 | -0.09 ^***^ | -0.01 | 0.01 | -0.01 | 0.517 / 0.664 |
|  | r | 0.21 ^***^ | 0.06 ^*^ | 0.01 | 0.00 | -0.12 ^***^ | -0.00 | 0.01 | -0.01 | 0.481 / 0.564 |
| **InfFroOrbGy** | l | 0.12 ^***^ | -0.01 | -0.01 | -0.00 | -0.05 ^*^ | 0.00 | 0.01 | -0.01 | 0.426 / 0.714 |
|  | r | 0.16 ^***^ | 0.01 | 0.03 | -0.00 | -0.09 ^***^ | 0.01 | 0.01 | -0.01 | 0.504 / 0.637 |
| **AntOrbGy** | l | 0.13 ^***^ | -0.00 | -0.01 | -0.00 | -0.00 | -0.02 | 0.01 | -0.01 | 0.377 / 0.688 |
|  | r | 0.12 ^***^ | -0.02 | -0.03 | -0.01 | -0.00 | -0.02 | 0.00 | -0.01 | 0.368 / 0.720 |
| **AntCinGy** | l | 0.45 ^***^ | 0.04 | -0.01 | -0.02 | -0.01 | -0.01 | 0.02 | -0.01 | 0.503 / 0.699 |
|  | r | 0.29 ^***^ | 0.09 | -0.02 | -0.02 | -0.09 | -0.00 | 0.02 | -0.00 | 0.312 / 0.703 |
| **MidCinGy** | l | 0.36 ^***^ | -0.01 | -0.07 | -0.01 | -0.19 ^***^ | -0.02 | 0.03 | -0.01 | 0.453 / 0.470 |
|  | r | 0.33 ^***^ | 0.08 | -0.02 | -0.00 | -0.27 ^***^ | -0.03 | 0.04 | -0.00 | 0.433 / 0.500 |
| **SupMedFroGy** | l | 0.55 ^***^ | 0.08 | 0.04 | -0.02 | -0.12 | -0.02 | 0.03 | -0.03 | 0.575 / 0.708 |
|  | r | 0.69 ^***^ | 0.05 | 0.03 | -0.04 | -0.06 | -0.04 | 0.02 | -0.05 | 0.557 / 0.675 |
| **SupFroGy** | l | 1.03 ^***^ | 0.06 | 0.06 | -0.04 | -0.03 | -0.08 | 0.01 | -0.05 | 0.495 / 0.699 |
|  | r | 1.02 ^***^ | -0.01 | 0.15 | -0.05 | -0.20 | -0.04 | 0.03 | -0.04 | 0.545 / 0.735 |
| **FroOpe** | l | 0.15 ^***^ | 0.05 | 0.03 | -0.01 | 0.01 | 0.01 | 0.01 | -0.01 | 0.269 / 0.405 |
|  | r | 0.16 ^***^ | 0.01 | -0.02 | -0.01 | 0.00 | 0.01 | -0.00 | -0.02 | 0.311 / 0.537 |
| **FroPo** | l | 0.23 ^***^ | -0.00 | 0.01 | -0.00 | -0.14 ^***^ | 0.01 | 0.01 | -0.00 | 0.476 / 0.681 |
|  | r | 0.25 ^***^ | -0.00 | 0.00 | 0.00 | -0.10 ^*^ | -0.01 | 0.02 | 0.00 | 0.451 / 0.699 |
| **MidFroGy** | l | 1.53 ^***^ | 0.18 | 0.04 | -0.05 | -0.34 | -0.04 | 0.09 | -0.00 | 0.538 / 0.587 |
|  | r | 1.56 ^***^ | 0.07 | -0.19 | -0.04 | -0.55 ^**^ | -0.08 | 0.08 | -0.05 | 0.572 / 0.634 |
| **MedFroCbr** | l | 0.15 ^***^ | 0.04 | -0.01 | -0.01 | -0.06 | 0.00 | 0.01 | -0.01 | 0.400 / 0.717 |
|  | r | 0.17 ^***^ | 0.01 | 0.00 | -0.01 | -0.01 | -0.00 | 0.02 | -0.01 | 0.410 / 0.670 |
| **AntIns** | l | 0.29 ^***^ | 0.03 | 0.02 | -0.01 | -0.01 | 0.03 | 0.01 | 0.00 | 0.469 / 0.622 |
|  | r | 0.30 ^***^ | 0.01 | 0.06 | -0.01 | -0.06 | 0.02 | 0.01 | 0.01 | 0.518 / 0.712 |
| **PosIns** | l | 0.16 ^***^ | -0.00 | -0.03 | -0.01 | 0.00 | 0.00 | 0.01 | 0.00 | 0.548 / 0.671 |
|  | r | 0.18 ^***^ | 0.04 ^*^ | 0.02 | -0.01 | -0.03 | 0.04 ^***^ | 0.01 | 0.01 | 0.535 / 0.639 |
| *Note.* * p<0.05 ** p<0.01 *** p<0.001; all values for fixed effects are betas; GM = gray matter volume; H = hemisphere; group = patient or healthy participants; cond. = condition (3D, 2D, E-book); Time = pre or post videogame intervention; Marg.R² = Marginal R²; Cond.R² = Conditional R²; N=136. | | | | | | | | | | |

**Blood biomarkers (BDNF, Interleukin 1 & 6)**

Biomarkers such as immunological markers Interleukin 1 and 6 pointing to peripherally measured inflammatory processes as well as the brain-derived neurotrophic factor (BDNF) have been associated with neuronal plasticity and psychiatric diseases [23, 24]. A meta-analysis by Green and colleagues [25] found a connection between reduced levels of BDNF (a biomarker also associated with synaptic plasticity) and schizophrenia. Animal models show that elevated levels of interleukin 6 in the hippocampus impeded long-term potentiation and neuronal plasticity [26, 27]. Given prior evidence, we therefore hypothesized that the concentration of those biomarkers may change as a function of the training intervention, precisely, we hypothesized a condition*time interaction for both groups (patients and healthy controls).

Methods. A blood sample (25 ml) per participant was taken by a medical professional directly before and after the training intervention including patients and healthy controls. BDNF determination was assessed using an ELISA kit (Biosensis Mature BDNF Rapid ELISA kit: human, mouse, rat; The barton, Australia). Plasma samples were appropriately diluted and detection of BDNF was carried out on a pre-coated mouse monoclonal anti-mature BDNF 96-well plate as described in the manufacturer’s protocol. The intra-assay and inter-assay coefficients of variation of this assay are below 10%. Samples were analyzed in duplicate, and mean values of respective measurements were calculated and used in statistical analyses. All measures were done in blinded fashion. Due to technical issues and possible defrosting of the samples during transport to the laboratory, the immunological parameters interleukin 1 and 6 could not be analyzed. The BDNF values were analyzed in exactly the same fashion as the MCCB behavioural data using a linear mixed model.

Results/Discussion. We did not find evidence for a training related change in BDNF concentration [Chi²(2)=2.59, p=.273]. Group was a significant predictor [z=-2.41, p<.05] indicating that patients with schizophrenia exhibit less BDNF concentration than healthy controls as has been shown before [24].

**Diffusion Tensor Imaging (DTI)**

A large proportion of studies show that schizophrenic patients have disorders of structural connectivity, e.g. Fasciculus uncinatus, cingulum fibers, the corpus callosum and/or the capsule internal [28, 29]. Hence, not only functional but also structural disconnectivity specifically between the HC and PFC has been suggested as an explanation for different groups of symptoms and their accompanying neurotransmitter dysfunctions in patients with schizophrenia [30-32]. For this reason, we wanted to explore whether structural connectivity changes as a function of the videogame intervention in schizophrenic patients but also in healthy controls.

Methods/Results. However, due to technical issues with the scanning protocol regarding the DTI sequence during data acquisition, we were not able to compute reliable statistics on this outcome measure.

# References

1. Kühn S, Gleich T, Lorenz RC, Lindenberger U, Gallinat J. Playing Super Mario induces structural brain plasticity: gray matter changes resulting from training with a commercial video game. *Mol Psychiatry.* 2014;19:265-71.

2. Gramann K, Müller HJ, Eick E-M, Schönebeck B. Evidence of Separable Spatial Representations in a Virtual Navigation Task. *Journal of Experimental Psychology: Human Perception and Performance.* 2005;31:1199-1223.

3. Nuechterlein KH, Green MJMMAI, Los Angeles, CA. MATRICS consensus cognitive battery. 2006;

4. Green MF, Nuechterlein KH, Kern RS, Baade LE, Fenton WS, Gold JM, et al. Functional co-primary measures for clinical trials in schizophrenia: results from the MATRICS Psychometric and Standardization Study. *Am J Psychiatry.* 2008;165:221-8.

5. Kay SR, Fiszbein A, Opler LA. The positive and negative syndrome scale (PANSS) for schizophrenia. *Schizophr Bull.* 1987;13:261-76.

6. Corrigan PW, Salzer M, Ralph RO, Sangster Y, Keck L. Examining the factor structure of the recovery assessment scale. *Schizophr Bull.* 2004;30:1035-41.

7. Cavelti M, Wirtz M, Corrigan P, Vauth R. Recovery assessment scale: Examining the factor structure of the German version (RAS-G) in people with schizophrenia spectrum disorders. *Eur Psychiatry.* 2017;41:60-67.

8. Kuznetsova A, Brockhoff PB, Christensen RHB. lmerTest Package: Tests in Linear Mixed Effects Models. *Journal of Statistical Software.* 2017;82:1–26.

9. Leucht S, Samara M, Heres S, Davis JM. Dose Equivalents for Antipsychotic Drugs: The DDD Method. *Schizophr Bull.* 2016;42 Suppl 1:S90-4.

10. Neyman J, Pearson ES. On the use and interpretation of certain test criteria for purposes of statistical inference. *Biometrika.* 1928;20A:175–240

11. Andersson JL, Hutton C, Ashburner J, Turner R, Friston K. Modeling geometric deformations in EPI time series. *Neuroimage.* 2001;13:903-19.

12. Whitfield-Gabrieli S, Nieto-Castanon A. Conn: A Functional Connectivity Toolbox for Correlated and Anticorrelated Brain Networks. *Brain Connectivity.* 2012;2:125-141.

13. Guy W. Clinical Global Impressions. ECDEU Assessment Manual for Psychopharmacology—Revised. Rockville, MD: US Department of Health, Education, and Welfare. *Public Health Service, Alcohol.* 1976;218-222.

14. Leucht S, Kane JM, Kissling W, Hamann J, Etschel E, Engel RR. What does the PANSS mean? *Schizophrenia research.* 2005;79:231-238.

15. Stephan KE, Baldeweg T, Friston KJ. Synaptic plasticity and dysconnection in schizophrenia. *Biol Psychiatry.* 2006;59:929-39.

16. Moghaddam B. Bringing order to the glutamate chaos in schizophrenia. *Neuron.* 2003;40:881-4.

17. Provencher SW. LCModel & LCMgui user’s manual. *LCModel version.* 2014;6:

18. Schwerk A, Alves FD, Pouwels PJ, van Amelsvoort T. Metabolic alterations associated with schizophrenia: a critical evaluation of proton magnetic resonance spectroscopy studies. *J Neurochem.* 2014;128:1-87.

19. Rothman DL, De Feyter HM, de Graaf RA, Mason GF, Behar KL. 13C MRS studies of neuroenergetics and neurotransmitter cycling in humans. *NMR Biomed.* 2011;24:943-57.

20. Ashburner J, Friston KJ. Unified segmentation. *Neuroimage.* 2005;26:839-51.

21. West GL, Zendel BR, Konishi K, Benady-Chorney J, Bohbot VD, Peretz I, et al. Playing Super Mario 64 increases hippocampal grey matter in older adults. *PLoS One.* 2017;12:e0187779.

22. Au J, Gibson BC, Bunarjo K, Buschkuehl M, Jaeggi SM. Quantifying the Difference between Active and Passive Control Groups in Cognitive Interventions Using two Meta-Analytical Approaches. *J Cogn Enhanc.* 2020;4:192-210.

23. Metti AL, Yaffe K, Boudreau RM, Simonsick EM, Carnahan RM, Satterfield S, et al. Trajectories of inflammatory markers and cognitive decline over 10 years. *Neurobiol Aging.* 2014;35:2785-2790.

24. Jones KA, Thomsen C. The role of the innate immune system in psychiatric disorders. *Mol Cell Neurosci.* 2013;53:52-62.

25. Green MJ, Matheson SL, Shepherd A, Weickert CS, Carr VJ. Brain-derived neurotrophic factor levels in schizophrenia: a systematic review with meta-analysis. *Mol Psychiatry.* 2011;16:960-72.

26. Frodl T, Amico F. Is there an association between peripheral immune markers and structural/functional neuroimaging findings? *Prog Neuropsychopharmacol Biol Psychiatry.* 2014;48:295-303.

27. Marsland AL, Gianaros PJ, Abramowitch SM, Manuck SB, Hariri AR. Interleukin-6 covaries inversely with hippocampal grey matter volume in middle-aged adults. *Biol Psychiatry.* 2008;64:484-90.

28. Wheeler AL, Voineskos AN. A review of structural neuroimaging in schizophrenia: from connectivity to connectomics. *Front Hum Neurosci.* 2014;8:653.

29. Canu E, Agosta F, Filippi M. A selective review of structural connectivity abnormalities of schizophrenic patients at different stages of the disease. *Schizophr Res.* 2015;161:19-28.

30. Meyer-Lindenberg AS, Olsen RK, Kohn PD, Brown T, Egan MF, Weinberger DR, et al. Regionally specific disturbance of dorsolateral prefrontal-hippocampal functional connectivity in schizophrenia. *Arch Gen Psychiatry.* 2005;62:379-86.

31. Scheel M, Prokscha T, Bayerl M, Gallinat J, Montag C. Myelination deficits in schizophrenia: evidence from diffusion tensor imaging. *Brain Struct Funct.* 2013;218:151-6.

32. Schmitt A, Hasan A, Gruber O, Falkai P. Schizophrenia as a disorder of disconnectivity. *Eur Arch Psychiatry Clin Neurosci.* 2011;261 Suppl 2:S150-4.
